# Real-Time Event-Based Unsupervised Feature Consolidation and Tracking for Space Situational Awareness

**DOI:** 10.3389/fnins.2022.821157

**Published:** 2022-05-06

**Authors:** Nicholas Ralph, Damien Joubert, Andrew Jolley, Saeed Afshar, Nicholas Tothill, André van Schaik, Gregory Cohen

**Affiliations:** ^1^International Centre for Neuromorphic Engineering, MARCS Institute for Brain Behaviour and Development, Western Sydney University, Werrington, NSW, Australia; ^2^Air and Space Power Development Centre, Royal Australian Air Force, Canberra, ACT, Australia

**Keywords:** event-based, tracking, space situational awareness, machine learning, neuromorphic, image processing

## Abstract

Earth orbit is a limited natural resource that hosts a vast range of vital space-based systems that support the international community's national, commercial and defence interests. This resource is rapidly becoming depleted with over-crowding in high demand orbital slots and a growing presence of space debris. We propose the Fast Iterative Extraction of Salient targets for Tracking Asynchronously (FIESTA) algorithm as a robust, real-time and reactive approach to optical Space Situational Awareness (SSA) using Event-Based Cameras (EBCs) to detect, localize, and track Resident Space Objects (RSOs) accurately and timely. We address the challenges of the asynchronous nature and high temporal resolution output of the EBC accurately, unsupervised and with few tune-able parameters using concepts established in the neuromorphic and conventional tracking literature. We show this algorithm is capable of highly accurate in-frame RSO velocity estimation and average sub-pixel localization in a simulated test environment to distinguish the capabilities of the EBC and optical setup from the proposed tracking system. This work is a fundamental step toward accurate end-to-end real-time optical event-based SSA, and developing the foundation for robust closed-form tracking evaluated using standardized tracking metrics.

## 1. Introduction

The near-earth space environment is an expansive but ultimately limited natural resource. This vantage point hosts a wide range of vital commercial, civil, and defence systems. As the space environment becomes more congested, the risk of collision increases, threatening a runaway rate of collisions that could render large regions of space unusable and be especially hazardous for crewed missions (Kessler and Cour-Palais, 1978). Although satellite motion models are well-understood, gradual changes to the orbit of RSOs occur due to atmospheric drag, collisions, unaccountable human error, deliberate actions, or other unexplained phenomena. Detecting and responding to these changes and anomalies in a timely manner is a vital step required to mitigating future collisions (Fujimaki et al., 2005). Space Situational Awareness (SSA) are critical techniques aimed at mitigating hazards to on-orbit systems by monitoring satellite traffic with accurate and timely data so we can continue to utilize the space environment in a sustainable way (Bobrinsky and Del Monte, 2010).

SSA operators use various systems to detect and observe RSOs actively or passively by sensing RSO emitted or scattered electromagnetic radiation across much of the electromagnetic spectrum. The performance and suitability of the various detection strategies, mostly in radar and optical regimes, differ in their ideal operating conditions, respective atmospheric opacity, detection limits, and the desired SSA task (Donath et al., 2010). Using an optical approach, SSA becomes a difficult vision task of high-speed target detection in low light, low Signal-to-Noise Ratio (SNR) conditions, with high accuracy requirements and complex scene dynamics due to stochastic atmospheric processes. However, with these challenges comes the advantage of intuitive data output, passive operation, and relatively low cost and power requirements compared to other techniques such as radar. As optical observation is inherently passive, the target or other observers are not directly aware of the observation. In contrast to active observation, the observer emits radiation to be scattered by a target which can then be detected. However, optical sensing techniques are inherently limited to observing during the night with clear weather conditions and are subject to atmospheric distortion.

Optical data collection acquires image frames using a telescope setup over a pre-determined exposure period to gather sufficient light to detect an RSO (Weeden et al., 2010). During these integrated exposures, a target RSO is carefully tracked by the telescope mount and control system to keep it within the Field-of-View (FOV) long enough to accumulate sufficient light for successful target detection. Collecting RSO position data from optical images is a well-studied detection problem of locating the center of an RSO from the target's apparent flux within the image frame (Zimmer et al., 2018). The position of the detected RSO and the time of the exposure can then be used for Orbit Determination (OD) to update or create the target's Two-Line Element Set (TLE) in a catalogue for future observation, ephemeris prediction, propagation, and ongoing satellite conjunction analysis (Ender et al., 2011).

**Figure 1:**
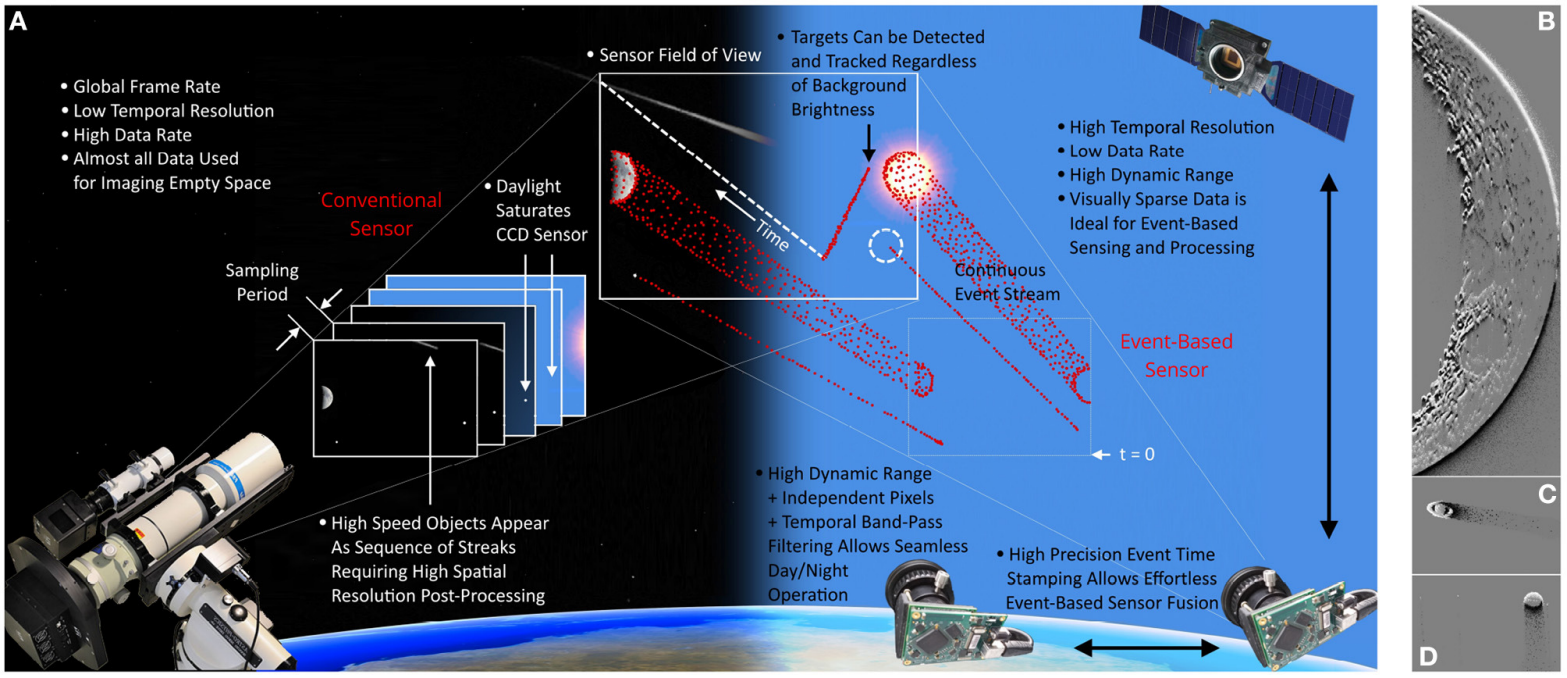
Comparison between conventional imaging **(A)**, event-based vision for space imaging, and SSA tasks. Sub-figures show the event-based output of the Moon **(B)**, Saturn **(C)**, and Jupiter **(D)** through a telescope. Figure from Afshar et al. (2020).

Recently, a new and unique optical imaging paradigm for SSA is emerging with the ongoing development of the neuromorphic EBC. These so-called “event-based” sensors make it possible to observe and track targets asynchronously while adapting to the visual dynamics of the scene. This is accomplished by leveraging the high temporal resolution, high dynamic range, low power and low latency of the EBC (Patrick et al., 2008; Gallego et al., 2019), as shown in Figure 1. Many of these advantages align well with the characteristics of SSA as an imaging task, which is highly sparse with the background sky being mostly free of stimulus, with exception to background point-like astrophysical objects such as stars. Recent research using EBCs for SSA have demonstrated and assessed the capabilities of EBCs mounted on terrestrial telescopes for imaging astrophysical objects and RSOs in various orbits (Cohen et al., 2019), in various observing conditions (Ralph et al., 2019), and the potential for SSA from on-orbit (Roffe et al., 2021).

Optical SSA using EBCs is a significantly different process to conventional optical SSA. Instead of exposing a sensor for a predefined period to accumulate light from a scene, an EBCs sensor array is always exposed to the scene, producing near-microsecond asynchronous binary contrast “events” whenever a pixel experiences a temporal change in light contrast. Such changes in illumination can be caused by the apparent motion of an RSO or atmospheric scintillation. This asynchronous event-based vision data cannot be used directly by conventional optical detection or tracking methods, given the EBC produces no frames and does not typically contain pixels that can measure light intensity. The consequence of the high temporal resolution and frame-free operation is that RSO detection becomes a problem of tracking a target in real-time as it moves within a telescope's FOV.

### 1.1. Conventional Target Tracking

Tracking is the process of locating a target within a scene successively in time. This process is a crucial component of many autonomous systems that comprises target detection, data association, and target state estimation (Blackman and Popoli, 1999). The general aim of tracking is to successively estimate the state of a target over time. This state is represented as a random variable and is modeled by observing it, or a sequence of other random variables associated with it, as observations (Wang et al., 2017). Tracking becomes a difficult task in the presence of an unknown and time-varying number of targets. Among these potential targets are also measurements which could be noise or clutter (Luo et al., 2020). The solutions in the conventional tracking literature vary, but are primarily based on mathematically rigorous methods with varying levels of statistical optimality. Most tracking methods use combinations of data association algorithms and state estimators, which resemble some recursive Markov-Bayesian process such as a Kalman or particle filter (Li et al., 2017).

Many Single Target Tracking (STT) and Multiple Target Tracking (MTT) algorithms have been developed. The simplest approach in basic applications is the Global Nearest Neighbour (GNN) tracker (Blackman and Popoli, 1999). GNN trackers perform state estimation by associating measurements to their nearest track based on a distance metric as the most probable data association. These techniques are very fast, simple to implement and MTT capable, but cannot correctly propagate the state and measurement uncertainty forward in time. This trade-off leads to GNN trackers being susceptible to noise and not properly accounting for the overall uncertainty of the track. This characteristic can lead to unrecoverable incorrect measurement-to-track association hypotheses (Smith et al., 2019). Many conventional computer vision trackers and neuromorphic trackers use the GNN for speed and simplicity, but at the cost of poorer track precision and less robust operation in the presence of noise and uncertainty.

The general solution to obtaining statistical robustness and precision are tracking and filtering algorithms that accommodate all measurements' hypotheses and respective uncertainties. Algorithms such as Probabilistic Data Association (PDA) (Bar-Shalom et al., 2009), Joint Probabilistic Data Association (JPDA) (Bar-Shalom and Tse, 1975), Multiple Hypothesis Tracker (MHT) (Blackman, 2004) and Probabilistic Multiple Hypothesis Tracker (PMHT) (Streit and Luginbuhl, 1995), solve this problem in varying ways by calculating all possible track hypotheses (including miss detection hypotheses) as distributions weighted by their respective time-varying likelihoods and measurement association probabilities. The PDA STT and JPDA MTT extensions are simple, but effective tracking algorithms of this class which have been shown to outperform the typical GNN tracking approach seen in many fundamental conventional and event-based tracking papers (Bar-Shalom and Li, 1995). Similarly, the MHT algorithm is a statistically optimal and more accurate MTT tracker than PDA and JPDA, but with a higher computational cost (Smith et al., 2019). These algorithms (particularly MHT), can quickly become intractable without significant hypothesis reduction because of the need to consider all possible track state hypotheses (Vo et al., 1999). This article aims to demonstrate how the event-based sensing paradigm has significantly different requirements and assumptions than conventional sensing, which can render simple trackers such as PDA highly effective.

Target models are also well-studied with point model architectures assuming a single-pixel size constraint on targets. Extended target models, however, can accommodate targets that can potentially produce multiple measurements at once (Granstrom et al., 2016). Extended tracking improves optimality with spatially distributed targets, especially with event data, since events do not necessarily represent a target's center of mass.

New development of novel filtering and estimation approaches have used algorithms such as Probability Hypothesis Density (PHD) (Mahler, 2003), Gaussian Mixture Probability Hypothesis Density (GM-PHD) (Vo and Ma, 2006), and Poisson Multi-Bernoulli Mixture (PMDM) filters (Mahler, 2014). These algorithms belong to a relatively new family of tracking and filtering methods based on Finite Set Statistics (FISST) which avoid explicit measurement-to-track association by representing tracks using random finite sets (RFSs) (Mahler, 2007b) and can be extended to model the number of trackable targets within the scene (cardinality) (Mahler, 2007a). While these algorithms present new and promising tracking alternatives to traditional Monte Carlo methods (Wang et al., 2017), they are beyond the scope of this article, as we focus here on exploring foundation methods of tracking in event-based sensing to formulate a statistically robust tracking framework from first principles.

Many of the foundation tracking publications detailed above use simulated data to evaluate their respective tracking algorithm. This approach helps to separate the accuracy and performance of the respective sensing system from the performance of the proposed tracker. Tracking performance with simulated and real-world data is evaluated using measures such as localization error, precision (Luo et al., 2020) and measurement-to-track assignment metric analysis such as Generalized Optimal SubPattern Assignment (GOSPA) (Rahmathullah et al., 2017).

While well-established tracking and state estimation algorithms exist in the conventional tracking literature, these algorithms are not directly applicable to event-based sensing. Conventional tracking algorithms applied to event-based data often require significantly more computational resources compared to event-based processing strategies since the former is not designed to correctly process the asynchronous, high temporal resolution, and event-driven output of the EBC.

### 1.2. Neuromorphic Event-Based Target Tracking

Event-Based (EB) algorithm design and raw data processing methods are often specifically tailored to particular objectives (Lakshmi et al., 2019). Currently, few EB algorithms exist for SSA purposes. New event-based algorithms are particularly challenging to design as the EB imaging paradigm is far removed from conventional frame-based vision. Various methods in the neuromorphic literature seek to represent and analyse event-based data in different manners, including using intervals of accumulated events into some frame as in conventional vision, or on the raw event-stream in a “neuromorphic” fashion. Algorithms designed to process EB data must be able to take full advantage of the benefits offered by the EBC, namely the high temporal resolution and asynchronous operation. A high-speed and accurate tracking algorithm “HASTE” (multi-Hypothesis Asynchronous Speeded-up Tracking of Events) (Alzugaray Lopez and Chli, 2020) has been recently shown to address many of these challenges. The authors explore the trade-off between rigorous state estimation and individual event processing while operating at real-time and on an event-by-event basis using a novel asynchronous patch-feature tracker that uses pre-learned feature templates. Pre-learned features are problematic for systems such as SSA, where it is desirable to operate without priors on the ideal spatio-temporal features that represent trackable targets since target appearance is rarely known and can vary based on tumble rate, illumination angle, material composition, atmospheric seeing conditions and orbital regime.

EB tracking and detection algorithms began with simple conventional computer vision detection of elementary shapes, and often used basic clustering or GNN-like strategies to associate events to tracks. These approaches included detecting and associating blobs (Delbruck and Lichtsteiner, 2007), lines (Everding and Conradt, 2018), Hough-transform extracted features (Ni et al., 2012), hand-crafted shapes, kernels and mean-shift (Lagorce et al., 2014), parts-based models (Valeiras et al., 2015). Broadly, these algorithms are all based on well-founded computer vision algorithms. A significant body of the literature also focuses on corner detection, employing well-established Harris and FAST key-point trackers (Alzugaray and Chli, 2018). Various optical flow estimation algorithms (Benosman et al., 2013) have also been proposed, which can be used to form the basis of a tracking algorithm. Machine learning approaches to tracking by detection have also been proposed, largely using GNN strategies or learned associations with supervised and unsupervised feature extraction for measurement detection (Lagorce et al., 2015; Afshar et al., 2019b). These algorithms operate on various representations of events, such as integrated frames, volumes (Wes Baldwin et al., 2021), graphs (Bi et al., 2020), and “time-surfaces” (Clady et al., 2015; Afshar et al., 2019a).

Many neuromorphic tracking algorithms utilize the Kalman filter or Markov-Bayes recursion for track state estimation. However, few use the previously described statistically robust trackers from the conventional tracking literature. Some examples of their use include the development of GM-PHD tracking (Foster et al., 2019) and MHT (Cheung et al., 2018). These algorithms operate on event clusters accumulated over time and accumulated frames of event-based data respectively. Additionally, GM-PHD has been designed to operate on normalized event-maps (Chen et al., 2020). A statistically robust tracker in the neuromorphic literature, also termed probabilistic data association, has been proposed (Zhu et al., 2017) as an expectation-maximization algorithm for optical flow, not to be confused with the established PDA tracker of the same name.

While many neuromorphic tracking algorithms are fast and accurate within the context of their respective applications, few are fully real-time or operate using the detailed state estimation techniques found in the conventional tracking literature. Although some of the articles discussed use Kalman filtering or a similar type recursion for track state estimation, track and measurement uncertainties are not fully taken into account without a more statistically robust solution such as PDA or JPDA. These algorithms are capable of accounting for all track hypotheses and fully propagate uncertainty into future time steps. Correct propagation of these uncertainties is key in SSA (Jones et al., 2015) since orbital uncertainty estimation underpins the effectiveness of many operational SSA activities such as orbit determination and conjunction assessment (Poore et al., 2016; Hilton et al., 2019). Statistically robust solutions aside, many trackers in the event-based literature also suffer poor robustness and adaptability to scene changes, with many hard-set and hand-tuned priors, and feature templates that are dependent on the scene or are task-dependent parameters. Many systems filter events, integrate events into structures (such as frames), or voxels, or do not fully characterize performance time for general application. Despite the variety of tracking approaches, learning-based feature detection and tracking methods also offer considerable “room for research” (Gallego et al., 2019).

### 1.3. Event-Based Space Situational Awareness

Early work in EBCs for SSA and astronomy has led to the development of event-based star trackers; devices typically used for spacecraft attitude estimation based on pattern recognition of background stars. Chin et al. (2019) leveraged the benefits of an EBC to perform high speed (although not real-time) and low power star tracking using a custom build data pipeline and a physically simulated star tracking dataset. Event-based SSA tracker systems have featured tracking algorithms which use elementary detection techniques such as Hough line detection on multi-scale fixed length streams of events as “chunks” (Bagchi and Chin, 2020) and detection *via* feature extraction on an event-by-event basis (Afshar et al., 2019b). Tracking has been performed using principled probabilistic MHT on frames of events (Cheung et al., 2018) at 25 Frames per Second (FPS) using Matlab. Using Poisson priors for the MHT requires 30 min to process 10 s of data using a Matlab implementation. Evaluations in Afshar et al. (2019b) and Cheung et al. (2018) were conducted using non-typical conventional tracking metrics, with Afshar et al. (2019b) using sensitivity and informedness and Cheung et al. (2018) with visual confirmation.

Despite the capabilities and novelty of the EBC, event-based SSA tasks are still a difficult vision problem. EBCs are noisy (Gallego et al., 2019), and when operating in low light conditions, EBCs produce a highly sparse event bandwidth in the order of 50,000 Events per Second (EPS). In especially difficult low-light conditions, noise levels can reach 400,000 EPS and the prevalence of “hot pixels” can become higher. Low-light blurring effects have also been observed as the result of delayed events known as “wake measurements” (Bar-Shalom and Tse, 1975) occurring behind targets in the scene, raising an “out-of-sequence measurement” problem. These challenges are exacerbated by the common practice of tuning EBC biases to maximize signal at the cost of increased noise levels. Conventional imaging techniques that integrate light over an exposure period often have a sensitivity advantage over EBCs since they can accumulate significantly more light and store intensity values at the pixel. Therefore, to use an EBC effectively for SSA, event-based observation systems and algorithms must take full advantage of the highly rich spatio-temporal information in the absence of intensity information. The combined challenge of processing event-based data properly and operating at a sufficient speed for SSA, is pushing the limits of the current EBCs and event-based algorithms. Few algorithms in the literature currently operate with the capabilities required to fully utilize an EBC for SSA. A clear trade-off exists between SSA capable tracking algorithms that use mathematically rigorous and accurate conventional Markov-Bayesian trackers with few tune-able parameters at high computational cost, and the high-speed low-cost computer vision style algorithms in the neuromorphic literature.

### 1.4. Contributions

In this article, we propose the FIESTA algorithm as a real-time and robust approach to event-based SSA tracking tasks. This algorithm addresses the challenges of processing the asynchronous and high temporal resolution output of the EBC accurately and efficiently. We successfully use an unsupervised approach to tracking-by-detection with few tune-able parameters and using concepts established in the neuromorphic and conventional tracking literature. FIESTA achieves real-time performance by operating asynchronously on the event basis and only utilizing events which represent potentially trackable targets detected by a novel feature consolidation algorithm. Using these strategies, the proposed algorithm can greatly reduce the computational and storage cost of a typical optical SSA system.

The functions and capabilities of FIESTA address one of the critical challenges in event-based sensing: the relevance of the individual event to tracking. We address this issue not through the integration of events using a static time interval or frame but instead using FIESTA to accumulate event information dynamically over time, in both the feature extraction and the tracking phase. We make full use of EBC data in a mathematically robust and closed-form solution to SSA state estimation by developing an asynchronous PDA algorithm. Using this algorithm, we explore the paradigm shift of event-based sensing embedded in an “interacting tracker-detector” feature consolidation system.

By exploring alternate approaches to unsupervised feature extraction and conventional interacting tracking, we developed the FIESTA algorithm with capabilities that support the SSA mandate of timely collection of accurate data on the space environment. We show that correct handling of high-temporal resolution and asynchronous EBC data, the asynchronous properties can lead to accurate RSO state estimation with a microsecond range latency even with a simple tracking algorithm such as PDA. Using FIESTA, we also show the EBCs capability to produce accurate “in-frame” (within the spatial foot-print of the pixel array) velocity estimation. This is usually a difficult SSA task using conventional image exposures which have limited temporal resolution. Although the EBC cannot measure absolute light intensity for spatial fitting, we leverage the high temporal resolution of the EBC to produce significantly more data points for position fitting than a single conventional image exposure. This proposed algorithm is the first example in the neuromorphic literature capable of unsupervised, robust and real-time tracking for SSA that leverages conventional and neuromorphic methods. In this article, We work to develop a foundation of conventional STT event-based tracking. Future work will involve developing additional tracking algorithms within FIESTA to perform a full suite of SSA tracking tasks with MTT capability.

## 2. Feature Extraction in Event-Based Sensing with FEAST

Tracking-by-detection is a common tracking method of detecting targets independently at each time step, then associating these targets over time to produce a track. An event-based detector or feature extractor for SSA tracking must handle the noisy output of the EBC in low light conditions, the characteristic high event rate and the low information capacity of an individual pixel. Event-based feature extraction using learning rules such as Spike Timing Dependent Plasticity (STDP) (Yousefzadeh et al., 2017) are often used to extract features while filtering noise. Feature Extraction using Adaptive Selection Thresholds (FEAST) (Afshar et al., 2020) is a simple and effective unsupervised feature extraction algorithm that has been previously used as for SSA tracking using the tracking-by-detection framework. FEAST runs on an event-by-event basis and can be used to extract salient features from the event-stream which may represent trackable targets. Based on hardware efficient models of STDP (Afshar et al., 2014, 2015; Sofatzis et al., 2014), the FEAST algorithm is a hardware-optimized model of feature extraction in a spiking neural network. Combined with a tracking algorithm, FEAST can be modified to remove the reliance on prior tracking information by detecting saliency and only tracking meaningful events, which significantly improves efficiency as only the detected salient events are processed by the downstream components of FIESTA.

### 2.1. Event-Based Vision Sensors

**Figure 2:**
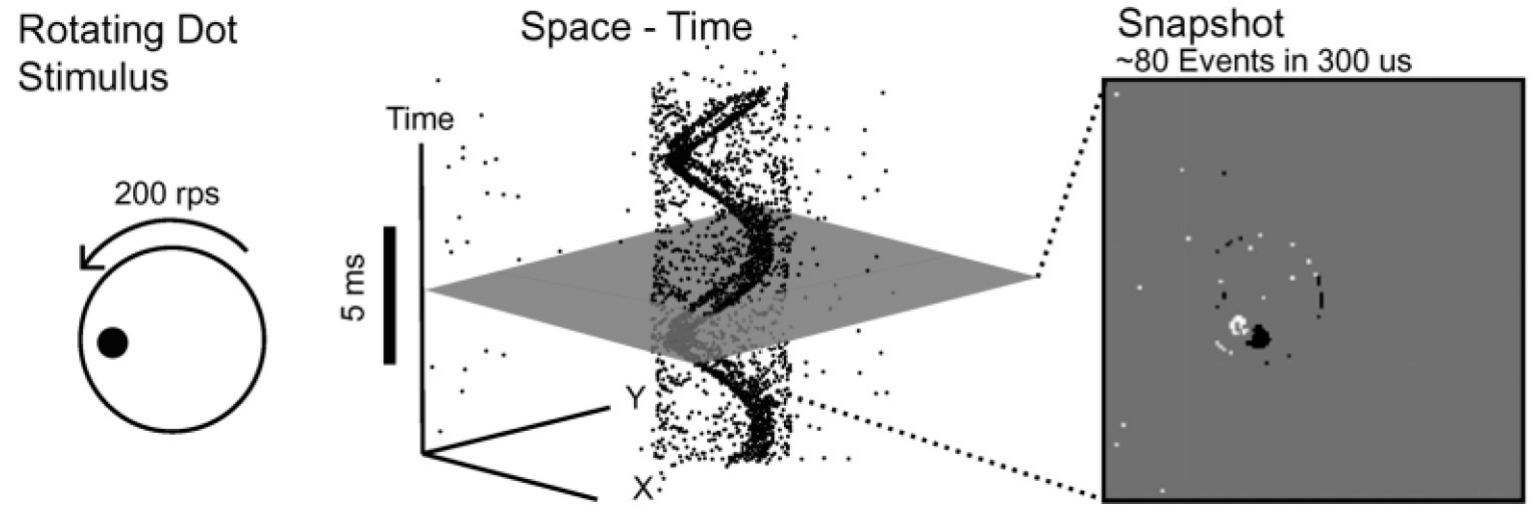
A demonstration of an event-based vision from Lichtsteiner et al. (2008), where a “rotating dot stimulus” will produce a continuous stream of events with high temporal resolution, where a frame-based system would instead produce image frames in discrete sample intervals.

The EBC was developed as an analogue model of the first stages of retinal processing, and built using neuromorphic principles (Delbruck and Mead, 1994; Patrick et al., 2008). In conventional imaging, sensors acquire a representation of the visual field as a frame of pixels acquired on a regular exposure interval. These frames are generated by synchronized integration at every pixels at a constant frame rate which are prone to blurring and saturation effects. The EBC, however, employs a drastically different imaging paradigm. Instead of producing image frames, the EBC uses an array of photo-receptors to produce representations of the visual scene from an asynchronous stream of “pixel events” triggered by logarithmic contrast changes detected at the pixel level (Lagorce et al., 2014), as shown in Figure 2.

The output of an event-based camera is a continuous stream of events *e*, with the following form:


(1) $$\begin{array}{l} e_{i}={\left[{\text{u}}_{i},t_{i},p_{i}\right]}^{T}\;i\in {\mathbb{N}}^{+}, \end{array}$$

Where **u**_*i*_ = [*x*_*i*_, *y*_*i*_] denotes the location of the pixel, *p* ∈ {+1, −1} the polarity of the change in illumination, and *t* the time at which the event occurred.

Adopting a similar notation to that used in Clady et al. (2015), we define the function Σ_*e*_ to map a time *t* to each 2D spatial coordinate **u**_*i*_:


(2) $$\begin{array}{ll} \Sigma_{e}: & {\mathbb{R}}^{2}\to \mathbb{R}\\ \;{\text{u}}_{i}: & t=\Sigma_{e}\left({\text{u}}_{i}\right) \end{array}$$

and a similar function *M*_*e*_ to map the polarity to each spatial coordinate:


(3) $$\begin{array}{ll} M_{e}: & {\mathbb{R}}^{2}\to \left\{-1,1\right\}\\ \;{\text{u}}_{i}: & p=M_{e}\left({\text{u}}_{i}\right). \end{array}$$

As time is inherently a monotonically increasing function, the function Σ_*e*_ defined in Equation (2) describes a monotonically increasing “surface”.

### 2.2. Time-Surface Calculation

A rich and descriptive feature representation called a “time-surface” (Delbruck, 2008; Afshar et al., 2019a) can be extracted from the event-stream to represent the features in the underlying visual scene.

Equations (2) and (3) create a 2D surface where τ defines the duration or “life-time” over which an event will have a non-zero value on the surface. The actual value for τ is a free parameter dependent on the application and nature of the visual field.

The exponentially decaying time-surface Π_*e*_(**u**_*i*_, *t*), weights the information carried by each event and is decayed toward zero over time. This surface is analogous to a high-pass filter:


(4) $$\Pi_{e}(u_{i},t)=\{\begin{array}{cc} M_{e}(u_{i})e^{\left(\frac{\Sigma_{e}(u_{i})-t}{\tau }\right)}, & \Sigma_{e}(u_{i})\leq t\\ 0, & \Sigma_{e}(u_{i})>t. \end{array}$$

The exponential time-surface has been shown to be more informative than the linear or the index-based time-surfaces (Afshar et al., 2019a). Newly calculated time-surfaces are normalized to make the system invariant to temporal scaling. This step re-scales the time-surface values from the original time-stamp values of the events on the surface. This normalization allows comparisons to be performed with other time-surfaces that may have occurred at a different time with different event time-stamps.

Time-surfaces are typically not calculated for the whole frame, but locally as an event-context centered on **u**_*i*_, given by $\pi_{e}^{r}\left({\text{u}}_{i},t\right)$ with a pixel radius *r*. We use an empirically determined event-context radius of 5, which produces an 11 × 11 time-surface event-context centered on the event. This is a practical choice due to the high computational cost required to calculate a time-surface for the full frame. When calculating an event-context with a fixed size, events which are too close to the edge of the sensor will encounter border issues and are discarded.

### 2.3. The Original FEAST Algorithm

The original FEAST algorithm functions as the basis for generalized classification or tracking systems. In these systems, FEAST is used to learn a series of salient features in an unsupervised manner, such as in k-means clustering (Lloyd, 1982). During classification or tracking, features within the scene are detected by matching them with selected prior learned features. FEAST, solely as a feature extraction algorithm, performs online unsupervised feature extraction using a clustering method with an adaptive selection threshold for each feature.

A FEAST network contains a layer of neurons *n*_*i*_ of total *N* neurons, with a feature representation (neuron weight *W*_*i*_) and an adaptive selection threshold, θ_*i*_. This threshold corresponds to the minimum similarity (cosine distance) required between the feature and a new incoming event-context for the neuron to update and learn. When a neuron update is triggered, that neuron is said to “spike”. This threshold is dynamic and varies based on two rules when the network is passed an event-context:

1. If the input event-context matches a feature *n*_*i*_ (the lowest cosine distance between its weights and the input event-context) and the similarity is within the feature's selection threshold θ_*i*_, the threshold is increased for feature *n*_*i*_ by a fixed amount Δ*i*. If multiple features match the input, the best matching feature is selected by the greatest cosine distance/similarity. Increasing this threshold raises the selectivity of the neuron (now inhibited) to future feature matching.
2. If an incoming event-context does not match given a feature *n*_*i*_, then all thresholds whose neurons matched to the event-context that were also not the best matching/winning neuron or within the selection threshold θ_*i*_, have their selection threshold's lowered by a fixed amount Δ*e*. Lowering this threshold reduces the selectivity of the neuron (now excited) to future feature matching.

In FIESTA with multiple layers, when a winning feature is successfully matched to a FEAST neuron, it spikes and generates an “event” for the next layer. The winning neuron learns the input event-context by updating the neuron feature weights with a fixed learning rate η as follows:


(5) $$\begin{array}{l} W_{n}=\left(1-\eta \right)W_{n}+\eta \pi_{e}^{r}\left({\text{u}}_{i},t\right), \end{array}$$

Where *W*_*i*_ denotes the weight of feature neuron *n*_*i*_, to which the input event-context $\pi_{e}^{r}\left({\text{u}}_{i},t\right)$ (of the wider time-surface Π_*e*_(**u**_*i*_, *t*)) is successfully matched. The FEAST algorithm is summarized in the Supplementary Materials.

The output of the network is in the form of $e_{j}={\left[W_{i},{\text{u}}_{i},t\right]}^{T}$ where *t* represents the time at which the original event occurred, that is, it remains unchanged from the input. Additionally, a flag is raised by the network to indicate that a given event-context caused a neuron to spike. The FEASTs strategy is to skip events that do not fall within a neurons distance threshold, which are therefore not yet considered informative to the current feature set, while still modifying neuron thresholds to increase receptivity. Under this learning rule, the number of feature events *j* will likely be lower than the total number of input events *i*. The output of FEAST is now significantly smaller than the full spatio-temporal feature space of the raw event-stream.

### 2.4. Online and Unsupervised Multi-Stage Feature Extraction and Consolidation in Real-Time

In this article, we demonstrate FIESTA as a novel and unsupervised feature consolidation algorithm to dynamically learn salient features occurring in the event-stream. This feature consolidation algorithm comprises a series of FEAST networks arranged as cascaded low-pass filters in a feed-forward multi-layer configuration. We use the spiking of FEAST neurons in FIESTA to indicate that a salient feature is developing at the spiking event's location. This salient feature may represent a trackable target and is, therefore, a reasonable input for a tracking algorithm. In our approach, we train and run FIESTA online and in real-time.

**Figure 3:**
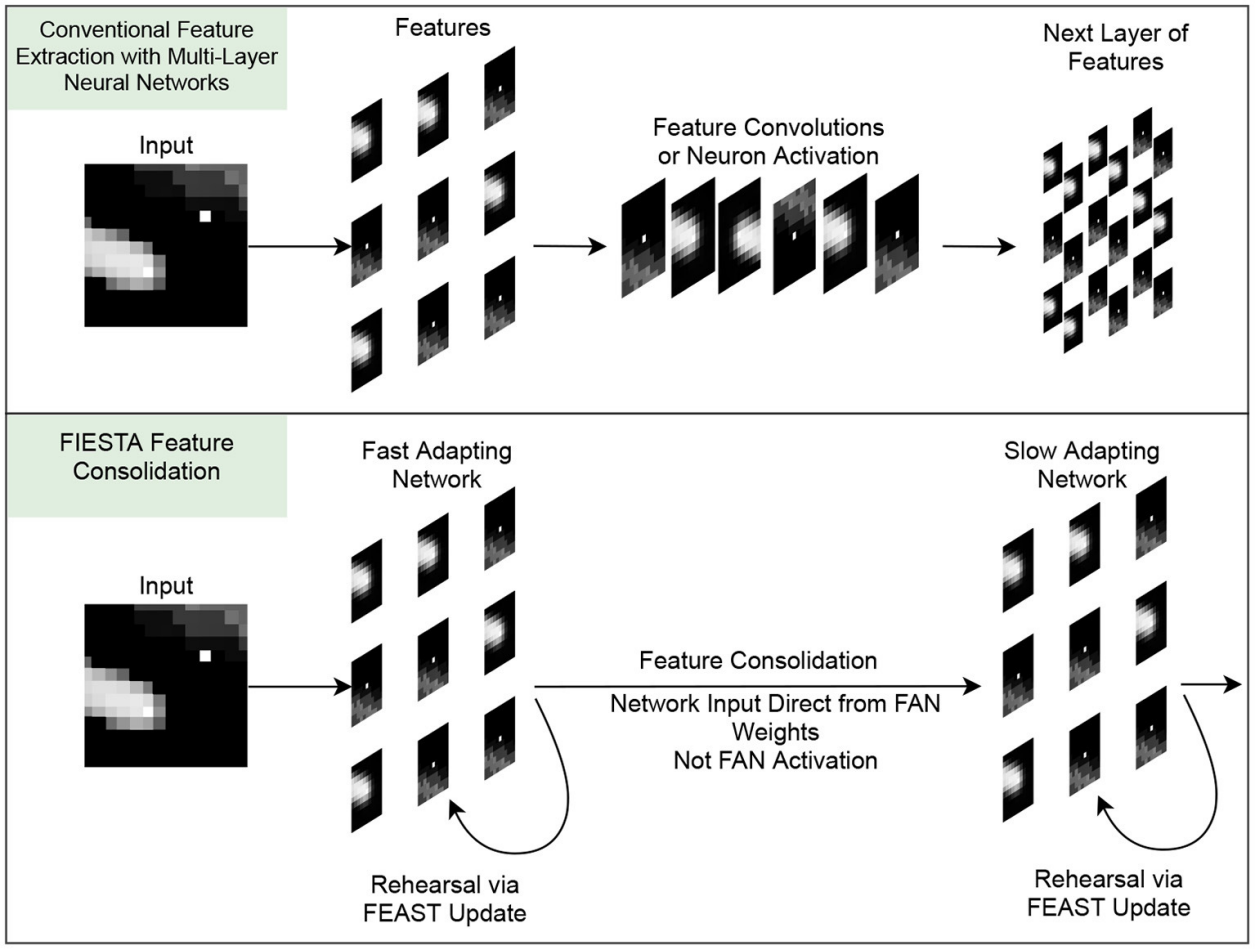
Comparison of conventional feature extraction and feature consolidation in FIESTA. In our novel feature consolidator, feature weights learned by the FAN are consolidated into the long-term SAN for later processing. This approach is as an alternative to the conventional approach of extracting features over multiple layers using feature convolutions or neuron activation.

To operate as an unsupervised and online tracking pre-processor with feature consolidation, FIESTA needs a balance between reacting to fast-occurring features while learning stable, slower developing features. This is difficult to achieve using a single FEAST network: slow learning rates will cause neurons to be react slowly whilst producing stable features. Alternately, high learning rates produce high plasticity and reactive extraction, but with a tendency to model poorer and less stable features. By taking inspiration from the workings of the hippocampus (Soldado-Magraner et al., 2020; Finnie et al., 2021), the balance between two such network behaviors can be struck by cascading feature extractors to form a feature consolidation network, as shown in Figure 3. The networks required in a feature consolidation network can be based on FEAST networks, but with different inputs and hyper-parameters. Combined with a simple activity measure filtering (discussed further in this section), these multi-stage FEAST networks form the feature extraction component of FIESTA. This alternate feature extraction approach significantly raises the effective SNR of the processed input event data by propagating the most salient spatio-temporal features to the tracker stage of FIESTA without the need for an explicit prior on the characteristics of trackable features.

The multi-stage feature extraction and consolidation network used in this article is comprised of two small FEAST networks containing only 9 neurons, and a single FEAST “track neuron” in a feed-forward arrangement. The first FEAST network is the Fast Adapting Network (FAN), and is comprised of neurons with a high learning rate to adapt to rapidly changing salient features and to constantly re-assign neurons with newly appearing features. If a neuron in this network spikes, the spiking neuron's weight is propagated to the second feature consolidation FEAST network, rather than activation as in conventional systems. This second network is the Slow Adapting Network (SAN) which learns slow-changing features from the FAN neuron weights instead of the event-stream. The tracker is given the event tuple of the current tracker update interval as the measurement of the current tracker index/scan *k* on the spiking of a SAN neuron and the SAN neuron weight.

In FIESTA, we aim to track the most salient feature without priors and in a less feature-specific approach to typical detectors. Along with FIESTA network activity, we assess the saliency and suitability of a feature by calculating the general “activity” of a feature. As shown in Equation 6, this activity measure is given by the sum of all elements of within an event-context or the winning neuron *n*_*i*_ weight matrix *W*_*i*_, and determining whether the activity sum is within the range of an activity threshold δ:


(6) $$\begin{array}{l} \underset{i}{\sum }W_{i}\geq \delta \end{array}$$

Although simple, this measure is unsupervised and sufficiently general to operate across multiple sensing environments. The computational simplicity of this method facilitates real-time operation. Additionally, this method simplifies the usual approach of having a prior on the “type” of feature to track. Instead, we focusing on “recent” activity and spatio-temporal feature structure as a proxy for saliency.

Similar activity, saliency and background noise filters are common tracker components in neuromorphic literature. Examples include band-pass filtering in the frequency domain (Scheerlinck et al., 2019), Leaky Integrate and Fire (LIF) neuron based filters of Surface of Activated Events (SAE) with frequency thresholding (Wan et al., 2021), spatio-temporal neighborhood filtering by examining the density of surrounding events (Feng et al., 2020) and biologically inspired multi-layered receptive fields for filtering and compression (Barrios-Avilés et al., 2018). While these methods each present many advantages, the focus of FIESTA is to reduce processing time and requires highly simplified filters. Such a filter needs to be computationally inexpensive, intuitive and capable of integration throughout FIESTA without any additional processing beyond the filter calculation itself. The simple summing operation of the proposed filter achieves this goal as a low cost operation that can be conducted on any event-context or neuron weight in FIESTAs various stages.

In FIESTA, the activity measure is first calculated for all event-contexts, and proceeding each FEAST layer in the feature consolidation network. Activity thresholding of the event-context removes contexts which contain too few recent events that likely represent a featureless noisy region or a region with independent and constantly spiking “hot-pixels”. In event-based SSA, bias settings are often tuned to raise the SNR of the event-stream at the cost of noise. These bias configurations often produce higher noise in low activity regions and increased prevalence of hot pixels. This practice further highlights the importance of a filtering mechanism in an event-based SSA algorithm.

## 3. Asynchronous Probabilistic Data Association Filtering and Tracking

The tracking component of FIESTA consists of a modified asynchronous PDA tracker (Bar-Shalom and Tse, 1975). This tracker accepts salient events as measurements from the FIESTA feature consolidator and operates in real-time, online, and unsupervised. We define an event as salient when the local event-context passes all filtering, activity thresholding, and spikes both the FAN and SAN. Here, we outline the principles of conventional tracking, the alternate paradigm of event-based tracking, and the proposed asynchronous PDA tracker.

### 3.1. Principled and Conventional Tracking

**Figure 4:**
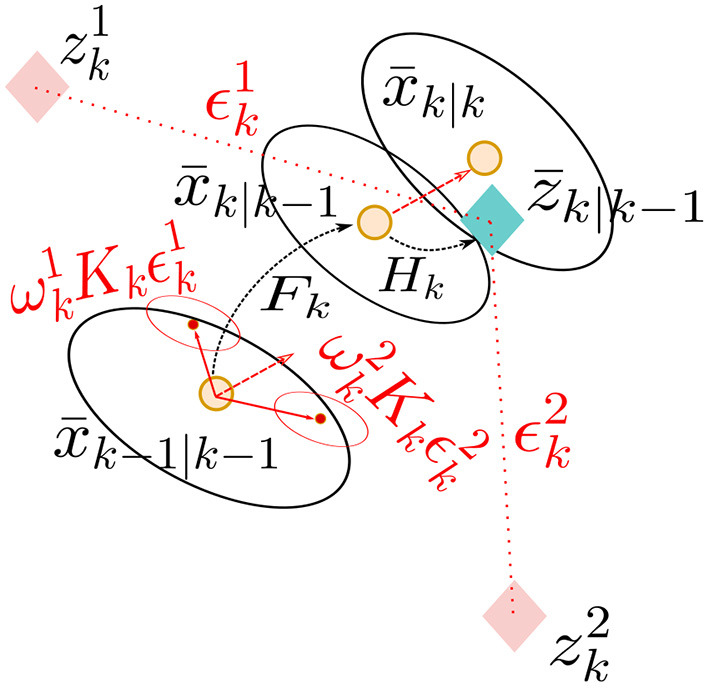
The tracking problem of estimating unknown target states *x*_*k*_, given incomplete measurements *z*_*k*_, at tracker time step *k*, in the presence of clutter measurements.

Tracking is a general assignment problem, where targets within a scene are associated to sensor measurements over time to form “tracks”. As shown in Figure 4, the tracking problem is a task of observing some unknown stochastic target state Probability Density Function (PDF) *x*_*k*_ at a discrete time step or “scan” *k*, given some imperfect sensor measurements or observations *z*_1:*k*_ within a measurement volume or FOV. Conventional trackers use assumed density filtering based on the Chapman-Kolmogorov Equation and Bayes' rule update to recursively refine the random target state estimate *x*_*k*_, given measurement information at each time step (Särkkä, 2013):


(7) $$\begin{array}{l} p\left(x_{k}|z_{1:k-1}\right)=\int p\left(x_{k}|x_{k-1}\right)p\left(x_{k-1}|z_{1:k-1}\right)dx_{k-1} \end{array}$$

(8) $$\begin{array}{l} p\left(x_{k}|z_{1:k}\right)=\frac{p\left(z_{k}|x_{k}\right)p\left(x_{k}|z_{1:k-1}\right)}{\int p\left(z_{k}|x_{k}\right)p\left(x_{k}|z_{1:k-1}\right)dx_{k-1}} \end{array}$$

By estimating these state densities, conventional filter-based trackers can model complex systems and how a target's state evolves over time. Trackers in the neuromorphic literature typically use either GNN type trackers or will operate without state estimators. These approaches do not optimally estimate target state or predictions densities. While computationally inexpensive, GNN tracker estimates a global hypothesis for target states by associating target measurements based simply on the closest spatial measurement or the most probable measurement association. State estimation is then conducted using this measurement-to-track association without propagating all system uncertainties or alternate hypotheses (such as miss-detection), into future time steps.

**Figure 5:**
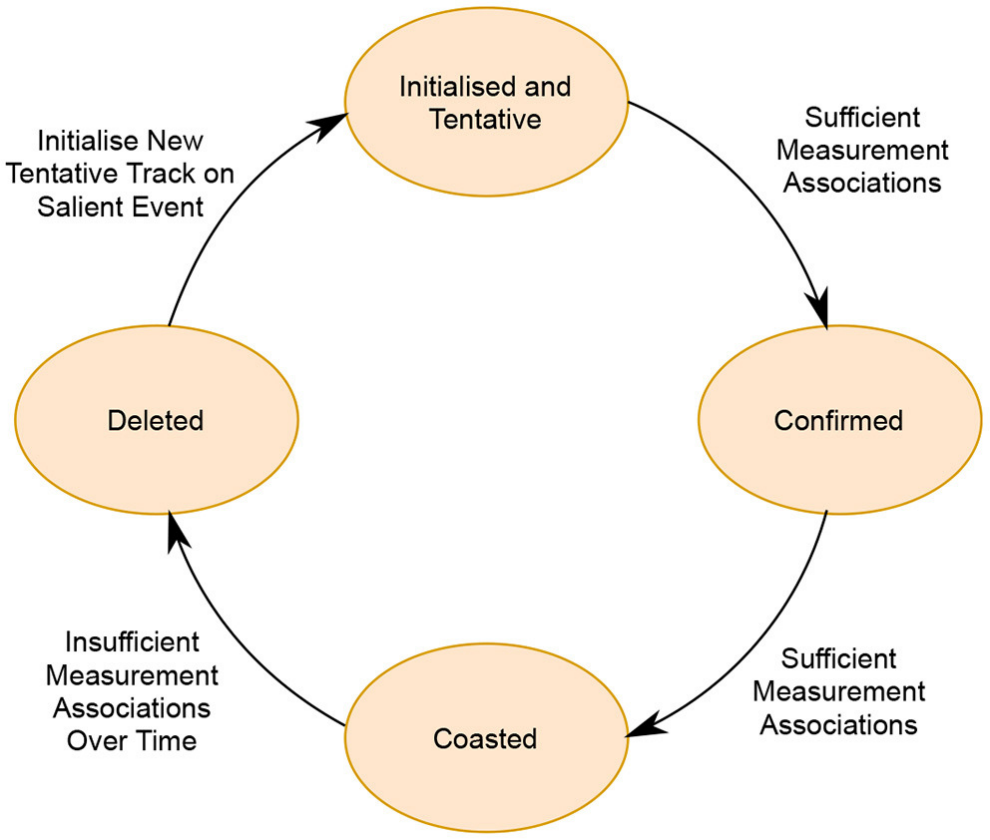
The established life-cycle of a track from initiation to deletion, as managed by track maintenance.

Modern trackers perform track maintenance to manage tracks and their life cycle throughout each time step, as summarized in Figure 5. In these track maintenance systems, tracks are first initialized when a new measurement is received by the tracker and it cannot be associated to any existing track. In this case, a new “tentative” or “un-confirmed” track is initialized. Tentative tracks could represent a true target, or a false target if it was associated to a “clutter” measurement or false alarm. A tentative track becomes “confirmed” once sufficient measurements or “evidence” is collected to confirm that it represents a genuine target. Without a measurement association in a given time step, a track is said to be “coasted”, where the track's state update is calculated based on its previous state prediction. If a track (confirmed or tentative) is not continually associated with sufficient measurements or becomes unlikely to represent a genuine target, it is deleted or “pruned”.

In tracking by detection, a detection process determines whether a given measurement originates from a target in the FOV and an association process determines which track a given measurement represents. For a measurement to be associated to a track, an assignment algorithm is used to build a set of data association hypotheses by calculating the assignment cost between all measurements at time *k* and all tracks *n*. This assignment task is a critical component of any conventional tracker and comprises a large part of the computational burden of tracking algorithms.

Broadly, “hard” Euclidean distance and “soft” elliptical/Mahanalobis distances are used to calculate assignment cost (Chen et al., 2002). Elliptical Mahanalobis distance association is the preferred approach as it bases association cost on the actual dynamics and uncertainties of the tracked target given the target covariance. This technique is contrasted to the less than ideal hard Euclidean distance calculation for assignment cost. Hard distance calculations are common place in neuromorphic literature due to their simplicity. This practice is also out of necessity since many such algorithms do not estimate state covariances, and cannot calculate soft distance costs as a result. Measurement gating is also often performed in the assignment step to identify and disregard spurious measurements (Wang et al., 2002). Measurements are usually gated if the assignment cost is too high and are unlikely to represent a true target.

The quantity and quality of measurement evidence accumulated by each track in the track manager can be calculated by various means, namely probability based logic, *m*/*n* (the ratio between the number of target associations and total measurements) history-based logic, and score-based logic. History-based assessment examines the number of measurement associations per total number of measurements over some time step interval. Probabilistic logic takes the likelihood of the track into account, where a low likelihood (high covariance) indicates a poor track. Score based logic is recognized as the best performing method (Blackman and Popoli, 1999) which combines metrics from target kinematics and task specific costs such as target intensity (in the case of optical systems) to generate a generic track logic score. In this STT version of FIESTA, we use history-based track logic due to the low computational cost, easily interpreted thresholds and simple implementation into the event index time step intervals used in FIESTA.

### 3.2. Conventional Tracking in the Event-Based Paradigm

Event-based sensing is a major paradigm shift in visual sensing and tracking. This shift necessitates several significant changes that conventional trackers must address to process event data optimally. These changes are caused by the asynchronous and parallel pixel read-out operations of the sensor. Events are only produced by activity changes in the scene with no guarantee of a new event at each time step. In some high activity cases however, the EBC can produce simultaneous events with the same time-stamp, but separate event indices ***e***_*i*_.

To appropriately handle event-based data, we track asynchronously by only updating tracks once an event is generated and when it passes the filtering and feature consolidator stages of FIESTA. This approach is fully event-based and efficient, since such a tracker processes updates proportional to the event-rate and the saliency of the global time-surface. Given the nature of the EBC and since FIESTA filters the event-stream, there is no guarantee that an identical Δ*t* occurs between each event after the filtering stage. Rather than defining a constant number of events or a constant time step over which to update the tracker state estimator, the tracker is updated as soon as a relevant feature is consolidated and extracted from the event-stream. Since state densities are updated for each event, a significant Δ*t* will result in state updates occurring inconsistently. These effects may not be noticeable, as the Δ*t* between each system time step *k* of the tracker is in the order of milliseconds in the worst case. It is important to note that the tracker system time step *k* (a convention in tracking literature) is the index of the events reaching the tracker after the feature extraction, not the event index of the event-stream which is the true temporal time step in terms of clock cycles. From here, we refer to the system time step *k* as the tracker update interval.

If a system can computationally afford to process each event individually, several assumptions and constraints greatly simplify calculations. By processing on the “event basis”, the assignment problem (for STT) becomes a one-to-one assignment, and in MTT just a one-to-many assignment. Abiding by this constraint in FIESTA, an association assignment is simplified to a one-to-one assignment, whereas with a STT algorithm, the single measurement per tracker iteration can only be assigned to a single track. The consequence is that a track may not produce a measurement event at each *k* in the presence of noise, which will cause the track to “coast” more often, where the track state is updated with it's own predicted measurement in the absence of a measurement from the sensor.

Tracking systems are designed with point or extended target models. The model of a point target assumes that target measurements contain no spatial information and will only produce a single measurement per *k* or tracker scan. Conversely, an extended target can produce multiple measurements which can contain spatial information. In FIESTA, we approximate event-based RSO tracking to point target models due to our assumption of one event per time step and due to the comparably low resolution of many current EBCs often leading to spatially diverse measurements falling on the same pixel. Ideally, RSOs would be tracked with an extended target model since RSOs are unresolved and often extended objects, with an unknown and varying Point Spread Function (PSF). This point target model does however treat all measurements as representations of the target center, despite most events originating from extended RSOs represent an object's flux rather than the target center. Improvements on this approximation will be investigated in future work with an extended object tracker.

### 3.3. Asynchronous PDA for Target State and Covariance Estimation

**Figure 6:**
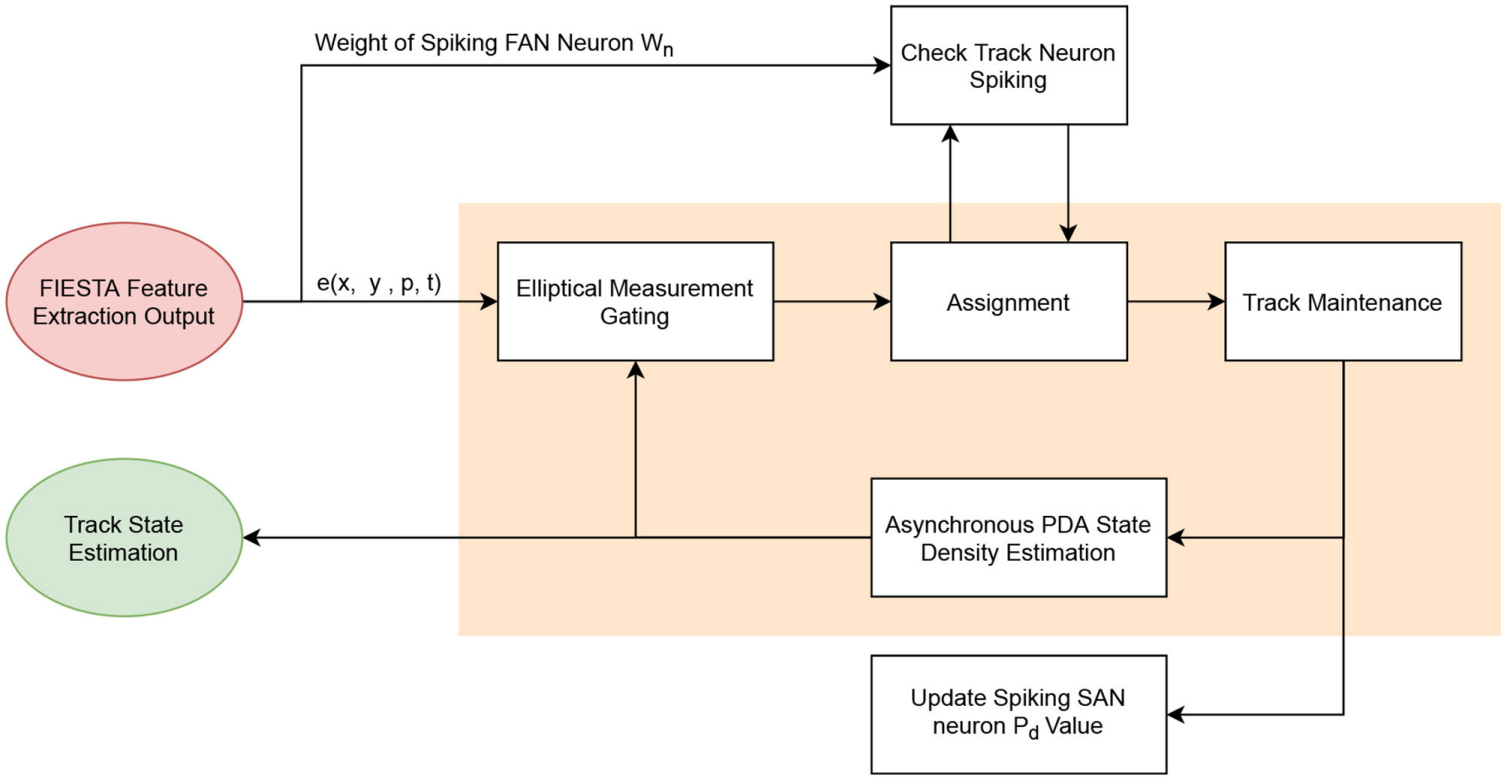
Outline of the proposed tracking component of FIESTA with the asynchronous PDA tracking recursion (orange highlight).

In FIESTA, we implemented an asynchronous variation of the PDA algorithm shown in Figure 6 to estimate the state and covariance of single point modeled targets in the FOV based on a events returned by the feature consolidator. PDA is a sub-optimal Bayesian STT algorithm that merges measurement association hypotheses weighted by association probabilities to calculate a target state update and prediction. This approach accounts for uncertainties in the estimated states and measurement associations, while also propagating them forward in time. For this reason, PDA operates significantly better than a simple Kalman filter or GNN tracker, in the presence of noise and clutter. The following initial assumptions are made in PDA (Bar-Shalom and Li, 1995):

- There is only one target to track within a given FOV,
- The track is represented by a density with a known mean and covariance (we assume linear and Gaussian),
- Measurements are independent and identically distributed with a uniform spatial distribution,
- Measurements are either an independently occurring true detection with probability $p_{d}^{k}$, or false alarms and clutter.

The proposed asynchronous PDA makes additional assumptions, as discussed in Section 3.1:

- Only one event can occur at a given step time *k*. Therefore there can only be a single measurement per tracker scan. This assumption is reasonable, although as mentioned, an EBC can occasionally produce two events with different indices with the same time-stamp,
- Tracker time steps *k* are asynchronous, only occurring when an event is received by the EBC and once the event has passed the feature consolidation and filtering stages. *k* here is monotonically increasing, non-uniform and limited by the time resolution of the EBC (~10μs in the best lighting conditions).

These asynchronous assumptions provide a more efficient means to process event-based data using conventional tracking by easing the computational burden of assignment. Outlined in Section 3.2, since we assume only one measurement per time step, assignment in STT and MTT cases become a task of one-to-one and one-to-*n* assignment, respectively. The trade-off, however, is that all tracks (one in our case) must be updated every time step. These updates are not as frequent as the time resolution of the system (or the EBC) since they occur asynchronously, and we only track events that have passed the feature consolidator. The implications of these assumptions are discussed in Section 6.

Since we assume linear and Gaussian models for our systems, we implement a special case of PDA where prediction and update steps can be calculated using Kalman filter recursion. The single target posterior PDF ρ, of the tracked target at step *k* given measurements from the first *k* to and including the *k* − 1 is expressed as a Gaussian density *N*, given by:


(9) $$\begin{array}{l} \rho_{\left(x_{k}|Z_{1:k-1}\right)}=N_{\left(x_{k};{\bar{x}}_{k|k},P_{k|k}\right)} \end{array}$$

where the estimated state mean ${\bar{x}}_{k|k}$, and covariance *P*_*k*|*k*_ is given by,


(10) $$\begin{array}{l} {\bar{x}}_{k|k}={\bar{x}}_{k|k-1}+K_{k}\left(z_{k}-{\bar{z}}_{k|k-1}\right) \end{array}$$

(11) $$\begin{array}{l} P_{k|k}=P_{k|k-1}-K_{k}H_{k}P_{k|k-1} \end{array}$$

where the Kalman gain is given by *K*_*k*_, and the measurement model as *H*_*k*_, which contains only terms for pixel positions. The predicted state mean ${\bar{x}}_{k|k-1}$ and covariance *P*_*k*|*k*−1_ is given by:


(12) $$\begin{array}{l} {\bar{x}}_{k|k-1}=F_{k}{\hat{x}}_{\left(k-1|k-1\right)} \end{array}$$

(13) $$\begin{array}{l} P_{k|k-1}=F_{k}P_{k-1|k-1}F_{k}^{T}+Q_{k} \end{array}$$

where *Q*_*k*_ is the process noise covariance (4 × 4 identity matrix in our case) and *F*_*k*_ is the state transition function for constant velocity:


(14) $$\begin{array}{l} F_{k}=\left[\begin{array}{cccc} 1 & 0 & \Delta t_{k} & 0\\ 0 & 1 & 0 & \Delta t_{k}\\ 0 & 0 & 1 & 0\\ 0 & 0 & 0 & 1 \end{array}\right] \end{array}$$

Δ*t* in the transition function *F*_*k*_ of the asynchronous PDA variation refers to the difference in the event time-stamp *t* of the previous *k* − 1 tracker time step and the current *k* tracker time step where:


(15) $$\begin{array}{l} \Delta t_{k}=t_{k}-t_{k-1} \end{array}$$

The predicted measurement estimate ${\bar{z}}_{k|k-1}$ is given as:


(16) $$\begin{array}{l} {\bar{z}}_{k|k-1}=H_{k}{\bar{x}}_{k|k-1} \end{array}$$

The state prediction and update is calculated with the Kalman gain *K*_*k*_ and covariance *S*_*k*_ at *k*. The predicted covariance *P*_*k*|*k*−1_ and measurement covariance *R*_*k*_ (2 × 2 identity matrix in our case) is given by:


(17) $$\begin{array}{l} S_{k}=H_{k}P_{k|k-1}H_{k}^{T}+R_{k} \end{array}$$

(18) $$\begin{array}{l} K_{k}=P_{k|k-1}H_{k}^{T}S_{k}^{-1} \end{array}$$

and the innovation ϵ_*k*_ given as:


(19) $$\begin{array}{l} \epsilon_{k}^{\theta_{k}}=z_{k}^{\theta_{k}}-{\bar{z}}_{k|k-1} \end{array}$$

All θ_*k*_ possible data associations used in PDA must be valid, where *m*_*k*_ is the total number of valid associations. In PDA filtering, these valid data associations are measurement-track associations that satisfy an elliptical gating threshold γ, where,


(20) $$\begin{array}{l} \epsilon^{\theta_{k}^{T}}S_{k}^{-1}\epsilon_{k}^{\theta }\leq \gamma \end{array}$$

In PDA tracking, the PDA state update is represented by a Gaussian mixture as the weighted sum of the mean of all valid measurement association hypotheses:


(21) $$\begin{array}{l} {\bar{x}}_{k|k}=\overset{m_{k}}{\underset{\theta^{k}=0}{\sum }}w_{k}^{\theta_{k}}{\hat{x}}_{i}^{k} \end{array}$$

and the estimated PDA covariance given by,


(22) $$\begin{array}{l} P_{k|k}=\overset{m_{k}}{\underset{\theta^{k}=0}{\sum }}w_{k}^{\theta_{k}}\left(P_{k}^{\theta_{k}}+\left({\bar{x}}_{k|k-1}-{\hat{x}}_{k|k}\right){\left({\bar{x}}_{k|k-1}-{\hat{x}}_{k|k}\right)}^{T}\right) \end{array}$$

where $w_{k}^{\theta_{k}}$ is the normalized hypothesis weight as the association probability normalized for all valid association hypotheses:


(23) $$\begin{array}{l} w_{k}^{\theta_{k}}=\frac{{\tilde{w}}_{k}^{\theta_{k}}}{\sum_{0}^{m_{k}}{\tilde{w^{i}}}_{k}} \end{array}$$

here, the unnormalized weight ${\tilde{w}}_{k}^{\theta }$ is given by,


(24) $${\tilde{w}}_{k}^{\theta }=\{\begin{array}{cc} 1-p_{D}^{k} & :\;\theta_{k}=0\\ \frac{p_{D}^{k}N(z_{k};{\bar{z}}_{k|k-1},S_{k})}{\lambda_{k}} & :\;\theta_{k}\in \left\{1,\ldots m_{k}\right\} \end{array}$$

The estimated mean state and covariance for each hypothesis is given by,


(25) $${\hat{x}}_{k}^{\theta_{k}}=\{\begin{array}{cc} {\bar{x}}_{k|k-1} & :\;\theta_{k}=0\\ {\bar{x}}_{k|k-1}+K_{k}(z_{k}^{\theta_{k}}-{\bar{z}}_{k|k-1}^{\theta_{k}}) & :\;\theta_{k}\in \left\{1,\ldots m_{k}\right\} \end{array}$$

(26) $$P_{k}^{\theta_{k}}=\{\begin{array}{cc} P_{k|k-1} & :\;\theta_{k}=0\\ F_{k}P_{k|k}F_{k}^{T}+Q_{k} & :\;\theta_{k}\in \left\{1,\ldots m_{k}\right\} \end{array}$$

In PDA filtering, λ_*k*_ is the clutter intensity as a Probability Mass Function (PMF) on the number clutter measurements expected within the measurement volume. λ_*k*_ is calculated by the size of the measurement volume *V*_*k*_ (resolution of the EBC in this case) and the expected clutter rate ${\bar{\lambda }}_{k}$:


(27) $$\begin{array}{l} \lambda_{k}=\frac{{\bar{\lambda }}_{k}}{V_{k}} \end{array}$$

Since we use a point model for our targets even while tracking an unresolved target, the state prediction will often jump perpendicularly toward the direction of motion toward the new measurements which occur on the so-called “wandering edge” of the target on its leading edge. This effect naturally leads to predictions that are nearly perpendicular to the target direction of motion. As a result, the predicted covariance and state in this PDA algorithm for a valid θ_0_, “no detection” hypothesis is given by the posterior covariance of the previous state, rather than the prediction of the previous state. This modification is shown above, where no detection hypothesis effectively assumes the target has stopped moving. This modification is reasonable for a no detection hypothesis in a sensor that measures contrast change occurring predominantly from motion.

We assume a constant velocity state transition model, as a “leap-frog” observed RSO (detailed more in Section 5) would move with a constant velocity through the stationary field unless it is recorded during a manoeuvre or other extenuating circumstances. We define our process noise covariance and measurement covariance as identity matrices, since we assume these covariances are Gaussian distributed with no correlations between the state variables and that the noise on the event-basis is relatively low with a unity variance.

The proposed asynchronous PDA has a maximum of two data association hypotheses. Since we track on an event-basis and only track single targets, these two valid hypothesis (*m*_*k*_ ≤ 2) are a miss-detection hypothesis $\theta_{0}^{k}$ and the true measurement association hypothesis $\theta_{1}^{k}$.

We experimentally selected a clutter rate ${\bar{\lambda }}_{k}$ of 1 × 10^−6^ and an initial ${p_{D}}^{0}$ of 0.75, which is tuned by the FIESTA network as detailed later in Section 3.4. These parameters place a moderate certainty in the probability of the EBC and feature consolidator to produce strong tracking candidates that represent actual target measurements, instead of clutter. These values become essentially irrelevant too close unity, requiring a miss-detection to be very close to the gating threshold before it can adversely affect the state estimation. This effect is more pronounced as the feature consolidator increases the *p*_*D*_ during tracking. Consequently, we have tuned the ellipsoidal gating and *m*/*n* track logic to prune tracks with poor likelihood, regardless of the *p*_*D*_.

In PDA, miss-detection hypotheses are penalized, and in FIESTA, tracks which are coasted for too long are deleted. By assuming a single measurement per scan, tracks are coasted (and penalized) more often with a miss-detection hypothesis. This tracker behavior is suitable since it is desirable to penalize a track that does not generate more events than clutter or background noise measurements. Discussed later in Section 6, this tracking approach may result in less optimal performance in a multi target scenario.

### 3.4. Tracker-Detector Feedback

In FIESTA, we include two feedback mechanisms for “tracker-detector” interaction. These interaction strategies allow the tracker to make decisions based on both spatial measurements from the event-stream and the spatio-temporal features of the tracked target. These mechanisms facilitate track-learn-detect style learning (Kalal et al., 2011). Here, the feature extractor gradually learns which modeled features are more likely to represent the spatio-temporal features of trackable targets.

The first tracker-detector interaction mechanism is a simple detection probability $p_{D}^{k}$ value association between SAN neurons. This mechanism encodes SAN neurons with a $p_{D}^{k}$ scaling factor based on how likely a SAN neuron is to model features that represent trackable targets. Since selection of a $p_{D}^{k}$ is non-trivial (Rezatofighi et al., 2015), we designed FIESTA to automatically tune the $p_{D}^{k}$ based on the tracker behavior and scene dynamics. By scaling the $p_{D}^{k}$ of a spiking SAN neuron, the hypothesis weight can scale the track state and covariance estimate by likelihood of whether the spatio-temporal features of the spiking SAN neuron represent a salient target detection. If the spiking event of a SAN neuron becomes associated with a track, and that track becomes confirmed, that spiking SAN neuron is then associated with a higher likelihood of producing confirmed tracks. Conversely, if a SAN spiking event is associated with a deleted track, the $p_{D}^{k}$ is lowered. We achieve this by associating a $p_{D}^{k}$ to each SAN neuron and tuning this $p_{D}^{k}$ by two factors *p*_*D*_+ and *p*_*D*_−, which raise or lower the neuron $p_{D}^{k}$ from an initial $p_{D}^{0}$. SAN neurons associated with coasted tracks are set to a mid-range $p_{D}^{k}$ value between the maximum and minimum $p_{D}^{k}$ (0.99 and 0.5 in our case). These penalties must lie within this range to allow neurons to recover from a deleted track and to prevent neurons consistently producing tracks with high $p_{D}^{k}$ near unity, even if the learned features no longer represent trackable targets.

The second feedback mechanism for tracker-feature extractor interaction in FIESTA involves the “track neuron”. This neuron facilitates an additional condition to the gating step before data association to discriminate between the spatio-temporal features of the track neuron and the current measurement. The track neuron is a FEAST neuron which gradually learns the features of a tracked target with similar hyper-parameters to neurons in the FAN. This neuron is updated with the weight of the SAN pre-synaptic FAN neuron. If the track neuron spikes and the neuron weight passes an activity filter, the current measurement association is considered a valid detection and the track is confirmed. This mechanism allows the track neuron to initiate track confirmation both when the appearance of the latest time context matches the learned appearance of the target, but also when the features of the tracked target appear to have sufficient contrast/activity. Additionally, this activity measure allows the track neuron to still function effectively in STT cases where the track neuron has no other tracks to differentiate.

Alongside the elliptical gating, this tracker-detector interaction allows FIESTA to correctly discriminate between measurements produced by noise/clutter or a true measurement based on their spatio-temporal features. The inclusion of the track neuron in FIESTA provides much needed spatio-temporal feature discrimination between track and measurement that PDA is only able to achieve based on the differences between their respective Gaussian and uniform densities. This strategy works to mitigate incorrect confirmation or association, where a measurement likelihood is high, but the event-context of the measurement has a different appearance to the track neuron's learned appearance of the tracked target. This track neuron is also asynchronous and operates on the event-basis by updating only when a track is successfully associated with a measurement.

## 4. The Overall FIESTA Algorithm

**Figure 7:**
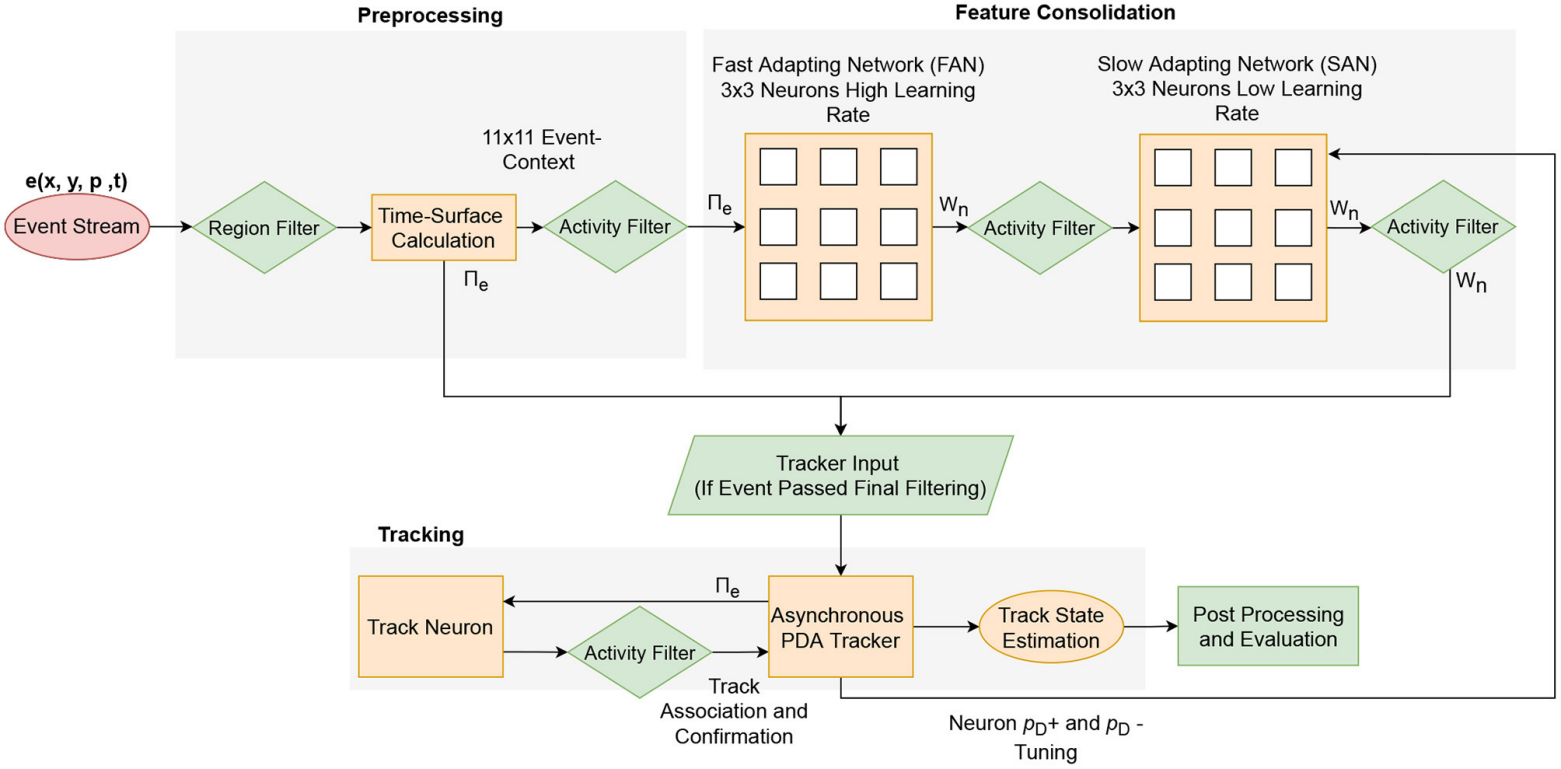
Outline of the overall FIESTA algorithm.

The proposed FIESTA algorithm has two main components, first feature extraction and then a tracker, as outlined in Figure 7. The tracking component of FIESTA consists of a modified asynchronous PDA tracker, which accepts events from FIESTAs multi-layer feature consolidator. The PDA tracker receives measurements as events **u**_*i*_ from the consolidator, only in the case it is considered salient, where the event's local event-context passes activity filtering and causes spiking in the FAN and SAN networks of the consolidator. The tracker uses a track neuron to gradually learn the appearance of the tracked target from the spiking FAN neuron of the current tracker interval, so associations can also be based on the spatio-temporal features of the current event-context and the tracked target. The detection probability *p*_*d*_ of a given event is tuned based on whether the currently spiking SAN neuron is associated to tracks that are frequently confirmed or deleted. The online learning and tracking components of FIESTA are reinitialized for each new observation in order to be robust to changes in the dynamics of the noise, scene, and targets between observations.

The FIESTA algorithm was written in C++, using the Eigen and OpenCV library. The asynchronous PDA tracking code was built onto a basic GNN tracking and Kalman filter framework (Konstantinova et al., 2003). All results were obtained using a PC with an Intel Core i7-7700HQ CPU @ 2.80 GHz × 8 with 16 GB of RAM. The PDA algorithm weight calculations were conducted in the log space.

FIESTA contains three main components, filtering, feature extraction and tracking. The feature consolidation stage of the system is comprised of two small FEAST networks in a feed-forward configuration. The first network is the Fast Adapting Network with a high learning rate to learn rapidly changing salient features and constantly re-assign neurons with different features. If a neuron in this network spikes, the neuron weight is propagated to the SAN which consolidates the slow-changing features from the FAN neuron weights. On spiking of a SAN neuron and the SAN neuron weight passing an activity threshold, the tracker is sent the event tuple of the current time step as the measurement of this time step.

Additionally, the spiking FAN neuron weight is propagated to the track neuron that belongs to the current associated track. FIESTA's strategy is to use the spiking of FEAST neurons to indicate that a salient feature is developing in the event-context at the location of the spiking event. This salient feature may represent a trackable target and is, therefore, a reasonable measurement input for the tracker. The tracker's output is parsed to a simple post-processor that filters events too close to the edge of the FOV and interpolates the tracker state positions in time using robust least-squares with bi-square weights. The interpolated outputs can now be ingested by a mission system, or in this case, our evaluation pipeline. The specific output of FIESTA in an operation mission system is observed state estimates of an RSO reported for multiple overhead passes (given range information is not measured), which can then be used in an OD workflow.

## 5. Results

In this article, we analyse a constrained STT case of so-called “leap-frog” event-based SSA data collection to develop theory toward real-time MTT. Leap-frog observing involves moving a telescope sidereally and observing an RSO as it passes through the FOV. This technique allows us to observe in a surveillance capacity for un-cued RSO detection or detection of an RSO with a known TLE. No other STT observing strategies are possible without background star motion which inherently requires a MTT approach. We examine this SSA data collection method as a means to evaluate FIESTA for STT. Similar to many conventional tracking papers, we use simulated tracking data to separate the accuracy and performance of a sensor from the tracking algorithm. We simulate targets observed using the sensor with the implementation described in Joubert et al. (2021), whose radiometric inputs are estimated based distortion-free point spread functions matching real-world observations of RSO at varying brightness with our telescopes. With this method, we create simulated leap-frogs of single targets transiting through the simulated FOV at random angles^1^ to separate the capabilities of the EBC and telescope setup from the performance of the proposed FIESTA algorithm.

Currently no real-world collected event-based SSA datasets exist exclusively for STT or the current generation of EBCs. Early datasets contain data collected with first generation EBC (Afshar et al., 2019b), possess vastly different performance and noise properties than the current third and fourth generation EBCs, and are not applicable to this algorithm.

We analyse nine different leap-frog observing scenarios. These scenarios include three different target brightnesses which we test at three different speeds, resulting in a total of nine scenarios, as detailed in Table 1. Each scenario contains 30 different simulations of a RSO entering the FOV at a random angle and position. Target speeds consistent with RSOs in Geostationary Earth Orbit (GEO) are not used to evaluate FIESTA in the leap-frog scenario since the combination of the leap-frog observing method and the low apparent speed of the RSO would produce very little detectable contrast for the EBC. The limiting magnitude of the current generation three Dynamic Vision Sensor (DVS) EBC has been observed to be approximately 13.5 using an 8-inch Riccardi-Honders (RH) Officina Stellare Telescope in a light-polluted suburban environment at the Western Sydney University, Werrington North Campus, Australia. Different optics, seeing conditions, and sky brightness are expected to result in a fainter limiting magnitude. The faintest targets examined here have a magnitude of 12 to ensure they would be within the limiting magnitude of the sensor, and could be reliably observed.

**Table 1:** Brightness and speed combinations for simulated RSO tracking cases.

**Case**	**Altitude (km)**	**Period (min)**	**Speed (pixel/s)**
Fast	200	98.5	1562.0
Medium	750	109.8	1342.0
Slow	2,000	127.2	1087.0
**Case**	**Faint**	**Medium**	**Bright**
Magnitude			
Brightness	12	9	6

A theoretical telescope and sensor configuration of a DVS346 on an 8-inch Officina Stellare was used to determine the pixel scale and speed of the simulations. This setup has been previously shown to successfully collect event-based SSA data (Cohen et al., 2019). This configuration has a wide FOV with a chip FOV of 37 × 28 arcminutes. Using the 346 × 240 pixel array of the DVS, the coverage of each pixel is 6.4 arcseconds in both dimensions.

We evaluate the accuracy of FIESTA by comparing the ground truth of the simulated dataset to the post-processed output of FIESTAs tracker. The tracker output constitutes the time-stamped state of all confirmed targets in the FOV each time an event reaches the tracker. This output is corrected and interpolated in the post-processor. The post-processor also removes track states within 20 pixels of the edge of the FOV. We perform this step to remove tracks that might remain in the FOV after the target has left, due to measurement updates from wake events. These wake measurements are also reported in the RADAR literature, where clutter measurements in the wake of a moving target are received by a tracking system due to spurious signal interactions with the target (Bar-Shalom and Li, 1995). In low-light event-based sensing, these effects can also be observed as a blurring effect (only in a conceptual sense, but still as out-of-sequence measurements), where pixels spike with a significant delay. These effects can be apparent in leap-frog recording and can cause a slight delay in track deletion as the target leaves the FOV but the track remains while still receiving measurements from the delayed wake events. Removing events close to the edge of the FOV largely eliminates the effects of the wake events. Sequential duplicated track state positions are also removed to mitigate error caused by the presence of hot pixels or wake events which cause affected track state to stall. Finally, track state positions are interpolated using robust linear regression with bi-square weights. This processed tracker output is then compared to the ground truth at each time step using the Matlab Sensor Fusion and Tracking Toolbox to calculate FIESTAs tracking performance.

**Figure 8:**
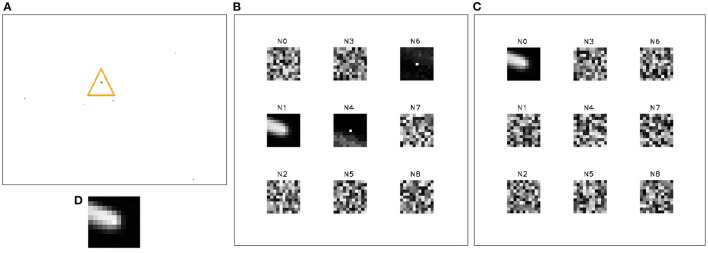
The output of FIESTA using a simulated “slow” scenario (2,000 km altitude) and medium brightness (magnitude 9) with the target moving from top left to bottom right corner **(A)**. In these results, FIESTA is shown to correctly track the target. The track neuron **(D)** learns the spatio-temporal features of the target. The FAN **(B)** and SAN **(C)** networks are behaving as expected, with the FAN learning fast changing features of the target in neuron N1, in addition to relatively noisy features such as the neurons N4 and N6 learned from event contexts surrounding the target. Here, the SAN learns fewer, but more rich and less noisy slower developing features from the FAN which best represent the salient target in the FOV in N0. Besides neurons N1, N4, N6 in the FAN and neuron N0 in the SAN, all other neurons have not been updated and remain in a random state.

**Figure 9:**
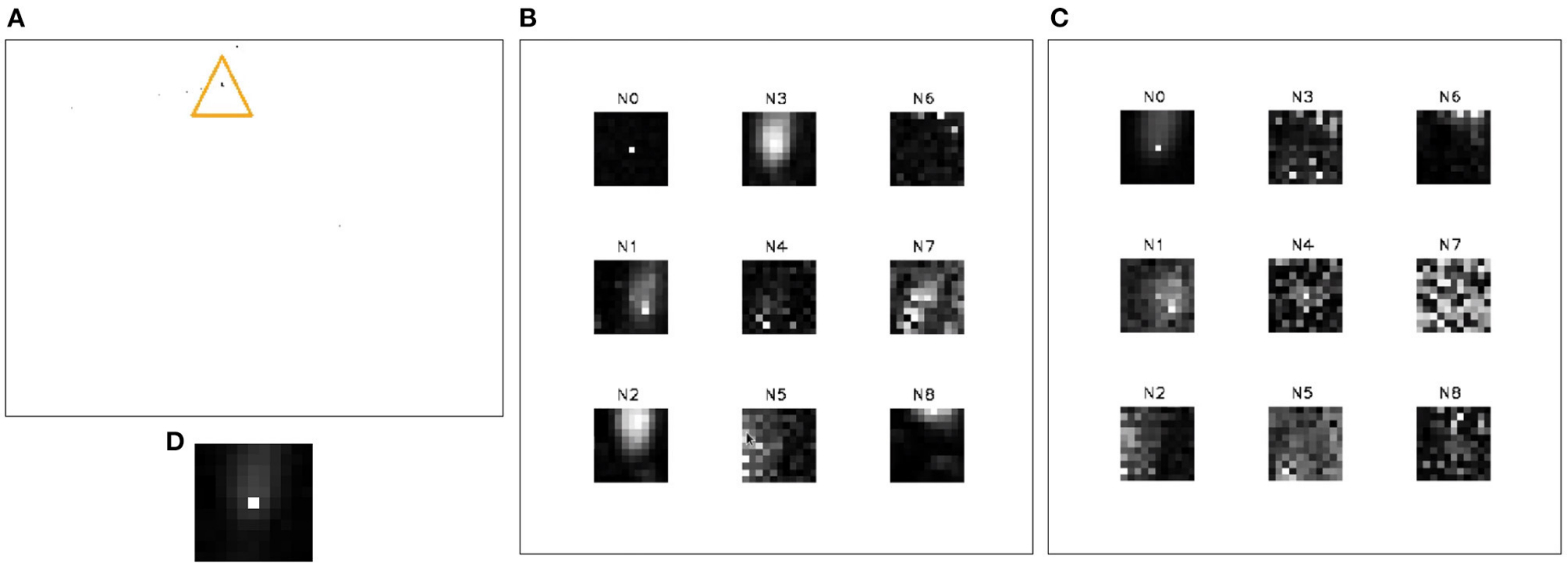
The output of FIESTA using real world observations of an unidentified RSO, collected using a DVS **(A)**. The tracking task is performed successfully and the feature consolidation neurons are behaving similarly to the simulated scenario with noisier features learned in the FAN **(B)** and fewer but more rich Q13 features learned in the SAN **(C)**. There are marginal differences in the noise properties between these two observations which has caused a fainter track neuron **(D)** and more noise neurons to be initialized in the FAN and SAN compared simulated scenario which is dominated instead by uninitialized neurons that have not learned any features. The only uninitialized neuron with a random state in the SAN is N7.

**Figure 10:**
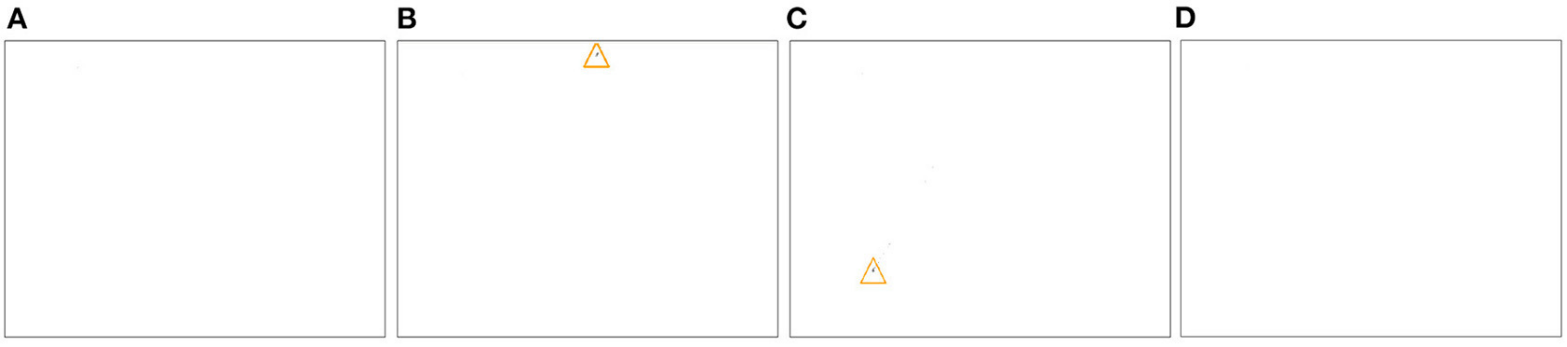
Leap-frog observation of RSO CZ-2C collected using a third generation ATIS EBC as it moves from the top of the FOV to the bottom. The observation is shown as a rendering of the event steam with a 200μs integration time. At time **(A)**, the target is moments from entering the FOV. At **(B)**, the target has appeared, FIESTA has learned the features of the target, has initialized and confirmed a track at the target location (orange triangle). At **(C)**, the target is leaving the FOV and at **(D)**, the target has left the FOV and most blur/wake events have disappeared.

**Figure 11:**
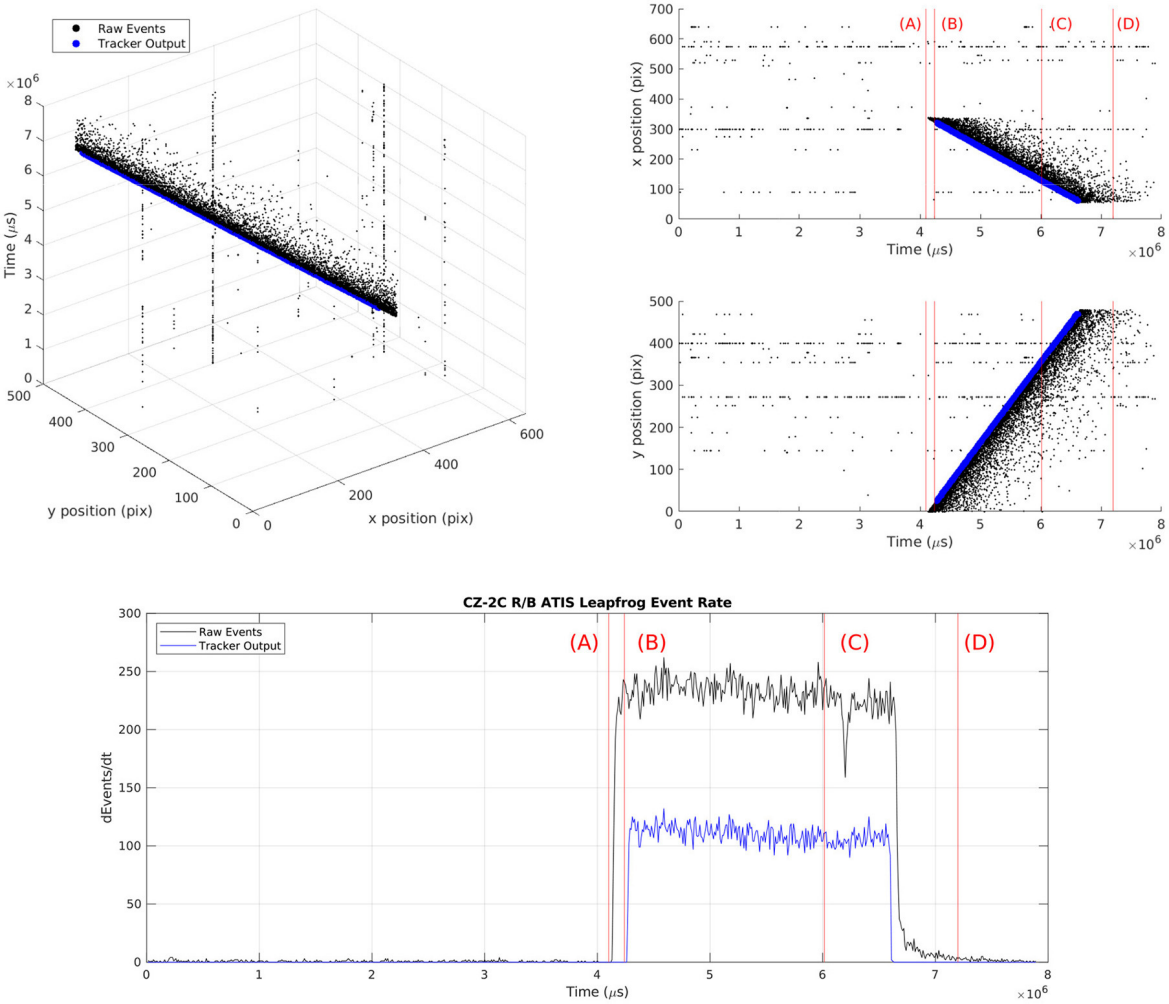
Scatter plots of raw events and tracker estimated position (top row) and the raw event rate and tracker event rate vs. time of the CZ-2C RSO leap-frog observation (bottom row) for each stage of the observation **(A–D)**. We show at **(B)**, that once the target has appeared, FIESTA initializes and confirms a track at the target location. By **(D)**, the target track has long ceased producing position outputs since the target left the FOV and it was deleted/filtered successfully despite the presence of wake events.

**Figure 12:**
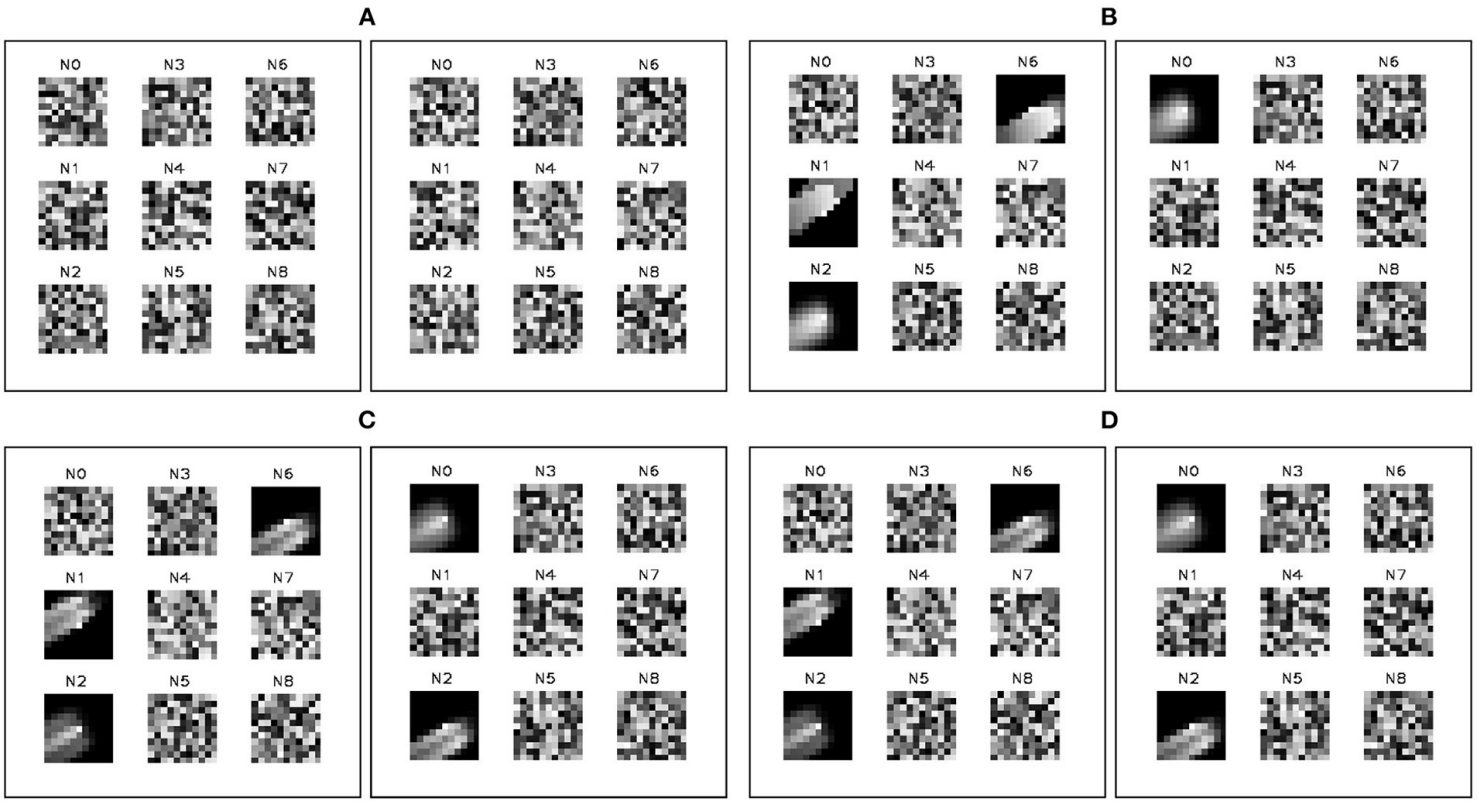
FIESTA features in the FAN (left panel of each sub-figure) and SAN (right panel of each sub-figure) learned during a leap-frog observation of RSO CZ-2C and shown at stages **(A–D)** of the observation. When the RSO enters the FOV **(A)**, the FAN rapidly learns fast changing, simple, and low resolution features of the target, while the SAN gradually learns richer, more stable, and longer term features of the RSO. The FAN and SAN features gradually refine into richer features and converge by **(C)**. When the RSO has left the FOV In **(D)**, the features are largely unchanged and inactive. These features will not be updated again until a new stimulus is present.

In Figures 8, 9, we compare the similarity of the FIESTA features learned from the simulated DVS to features learned from real-world collected event-based data. We show FIESTAs feature consolidation and tracking behavior on a leap-frog observation of RSO CZ-2C R/B using a third generation ATIS EBC in Figures 10–12. Successful tracking in these figures highlight the robust nature of FIESTA using different sensors without any parameter changes. These figures demonstrate FIESTAs low latency with a short 250*ms* delay between the target's first appearance in the FOV and the confirmation of a track centered on the new target. This latency is in part affected by the post-processor which is removing tracks which occur too close to the FOV as previously discussed. The post-processor is also responsible for the track being deleted just before the RSO leaves the FOV.

The differences between the raw event rate and the tracker state estimation rate indicates FIESTA is successfully disregarding a significant portion of the events as clutter. These figures show FIESTA correctly handles the asynchronous nature of EBC data where feature learning and tracking occur only when sufficient salient events are produced. In Figure 11, this asynchronous operation is also apparent with no learned features before the RSO enters the FOV at time (A) and with no changes in the network features after the RSO has left the FOV at time (D). Blur events and hot pixels are particularly apparent in the comparison of the plotted track position and the raw events. These hot pixels are shown to not affect tracking.

We quantify the performance of FIESTA by calculating the error between the state estimation output of the tracker and to the simulated ground truth state of targets in Root Mean Squared Error (RMSE). Additionally, we examine the latency of FIESTA with the average time per event and the average time required to acquire a target once it enters the FOV and a confirmed track is initialized. Finally, we compute the GOSPA metric for each simulated track scenario. GOSPA jointly estimates the localization error of confirmed true targets and an assignment performance based on the number of correctly detected, missed and false targets (Rahmathullah et al., 2017). Although GOSPA is less informative for STT cases than MTT, we calculate the mean GOSPA for each scenario to produce a metric which jointly describes the assignment and localization error.

**Figure 13:**
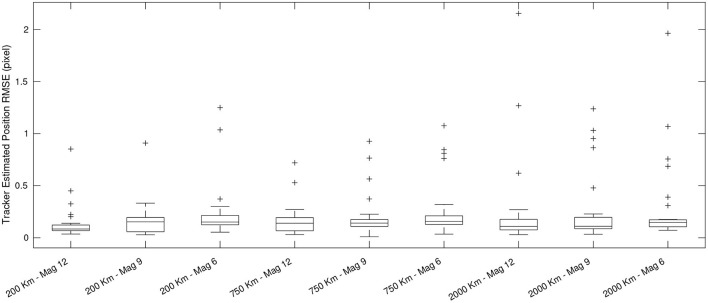
Comparison of the RMSE localization error (pixel) recorded in each of the nine simulated RSO altitude and magnitude brightness scenarios. In each scenario, 30 different recordings were evaluated.

We observe a consistent sub-pixel average localization error and variance across the evaluated simulations in Table 2 and Figure 13. The best performance is obtained on the fastest scenarios where the simulated target has a higher contrast and produces more events. The lowest performance was found in the slower and fainter scenarios where fewer events are produced by the target due to lower contrast. The lowest error recorded was on the fastest (200 km altitude) simulations. When comparing the magnitude 9 and magnitude 6 brightness results across all altitude cases, the error is shown to increase. This is due to targets becoming less point-like (as our target model assumes) and exhibiting more extended structure as the brightness increases, which results in poorer performance as events cannot be assumed to represent the target center of mass.

**Table 2:** Localization and track assignment metrics recorded across each testing scenario.

**Case altitude and brightness**		**Average position** **RMSE (pixel)**	**STD position** **RMSE (pixel)**	**Average velocity** **RMSE (pixels per Second)**	**STD velocity** **RMSE (pixels per Second)**	**Average** **GOSPA**	**STD** **GOSPA**
200 km	Mag 12	0.200	0.224	1.608E-06	4.599E-06	0.178	0.043
	Mag 9	0.158	0.125	7.687E-07	1.467E-06	0.145	0.062
	Mag 6	0.204	0.158	5.436E-07	9.342E-07	0.186	0.076
750 km	Mag 12	0.330	0.292	5.393E-07	1.105E-06	0.307	0.042
	Mag 9	0.188	0.103	6.967E-07	1.204E-06	0.172	0.060
	Mag 6	0.235	0.282	1.476E-06	4.012E-06	0.194	0.078
2,000 km	Mag 12	0.320	0.189	5.927E-07	1.162E-06	0.305	0.062
	Mag 9	0.202	0.103	7.026E-07	1.565E-06	0.186	0.074
	Mag 6	0.262	0.157	9.557E-07	2.450E-06	0.244	0.078

The velocity estimation error is found to be very low and consistent with the low recorded localization error. A small number of outliers are observed in the faintest and lowest contrast simulations, as shown in Figure 13, represented by crosses. The error of these outliers is within 2.2 pixels. Analyses of each case show the outliers are the result of fitting errors in the post-processor or the FIESTA failing to detect sufficient events for a more accurate and continuous state estimation, which often results in momentary track switches.

In Table 3, the velocity and localization errors are expressed in arcseconds. Our angular projection is given by the pixel scale of the DVS364 in the optical setup described in Section 5, which is 6.4 arcseconds per pixel. Our recorded localization error is observed to be consistently within 4 arcseconds. Shown in Table 4, the highest horizontal error is 170.416 m at 2,000 km altitude with a localization error of 1.758 arcseconds and a horizontal error of 8.510 m in the best case with an altitude of 200 km at 0.878 arcseconds of localization error. The recorded error is within a suitable range to perform accurate OD on the simulated targets. Given a higher resolution EBC and optical setup with a smaller FOV, the individual pixel coverage can be reduced to provide higher accuracy RSO state estimates.

**Table 3:** Localization and track metrics recorded across each testing scenario expressed in arcseconds based on the pixel coverage of the DVS364 in the optical setup described in Section 5 at 6.4 arcseconds of coverage per pixel.

**Case altitude and brightness**		**Average position** **RMSE (arcsec)**	**STD position** **RMSE (arcsec)**	**Average velocity** **RMSE (arcsec per Second)**	**STD velocity** **RMSE (arcsec per Second)**
200 Km	Mag 12	0.878	1.031	2.792E-05	7.676E-05
	Mag 9	1.039	1.031	1.987E-05	7.378E-05
	Mag 6	1.474	1.654	3.948E-05	1.282E-04
750 Km	Mag 12	1.052	0.920	5.899E-06	1.352E-05
	Mag 9	1.280	1.308	1.584E-05	3.349E-05
	Mag 6	1.614	1.650	2.812E-05	6.322E-05
2,000 Km	Mag 12	1.523	2.757	1.852E-05	5.915E-03
	Mag 9	1.557	2.080	2.562E-05	5.722E-05
	Mag 6	1.758	2.504	3.479E-05	9.369E-05

**Table 4:** Localization and track metrics recorded across each testing scenario expressed in horizontal error (m) based on the pixel coverage of the DVS364 and the selected optical setup at 6.4 arcseconds of coverage per pixel and the RSO altitude at each test scenario.

**Case altitude and brightness**		**Average horizontal position error (m)**	**STD horizontal position error (m)**	**Average horizontal velocity error (m per Second)**	**STD horizontal velocity error (m per Second)**
200 Km	Mag 12	8.510	9.994	2.707E-04	7.443E-04
	Mag 9	10.072	9.997	1.927E-04	7.154E-04
	Mag 6	14.295	16.041	3.828E-04	1.243E-03
750 Km	Mag 12	38.265	33.470	2.145E-04	4.914E-04
	Mag 9	46.538	47.543	5.760E-04	1.218E-03
	Mag 6	58.685	59.993	1.022E-03	2.299E-03
2,000 Km	Mag 12	147.678	267.293	3.373E-03	9.084E-03
	Mag 9	150.986	201.673	2.485E-03	5.549E-03
	Mag 6	170.416	242.838	3.373E-03	9.084E-03

Average GOSPA scores (as a cost between 0 and 1, where 0 indicates no error) indicate that the localization error is the most dominant error component of FIESTA, thereby demonstrating robust assignment behavior. False tracks were not observed in any simulation. Track switching, however, is relatively common and occurs on average once per recording across all recording cases. The standard deviation of all recorded performance metrics is low, indicating the system is robust and stable across all dataset recordings.

As detailed in Table 5, the processing time for each simulation across all tested scenarios was faster than real-time, with events being processed under 40 μs. These speeds are shown to be independent on the event bandwidth. This is likely the result of FIESTAs feature consolidator dynamically tuning the amount of processing it performs by assessing the information content of events that might represent trackable data, rather than processing all events indiscriminately. Time performance per event is evaluated as the average time taken to process all events in the event-stream, regardless of whether the event reached the tracker or was filtered. High bandwidth simulations in order of 25,000 - 41,000 events per Second were still processed in real-time, which clearly demonstrates FIESTAs efficiency and low latency.

**Table 5:** Computational performance metrics recorded across each testing scenario.

**Case altitude and brightness**		**Average bandwidth** **(events per Second)**	**STD** **Bandwidth** **(events)**	**Average time** **per event (us)**	**STD time** **per event (us)**	**Average** **real-time (%)**	**STD** **real-time (%)**
200 Km	Mag 12	38137.859	10.200	3.253	730.597	314.587	31.695
	Mag 9	18442.608	10.867	2.488	522.433	136.346	19.108
	Mag 6	14537.104	11.500	1.996	380.998	94.154	23.819
750 Km	Mag 12	19243.195	10.333	2.023	642.590	144.932	29.298
	Mag 9	20073.603	10.133	2.193	548.930	139.189	21.760
	Mag 6	19970.590	11.033	2.236	404.956	113.422	15.892
2,000 Km	Mag 12	20050.127	9.300	1.932	712.197	160.545	25.667
	Mag 9	16992.072	10.333	2.040	519.937	114.501	21.081
	Mag 6	19551.495	10.300	2.120	434.300	121.547	28.676

**Figure 14:**
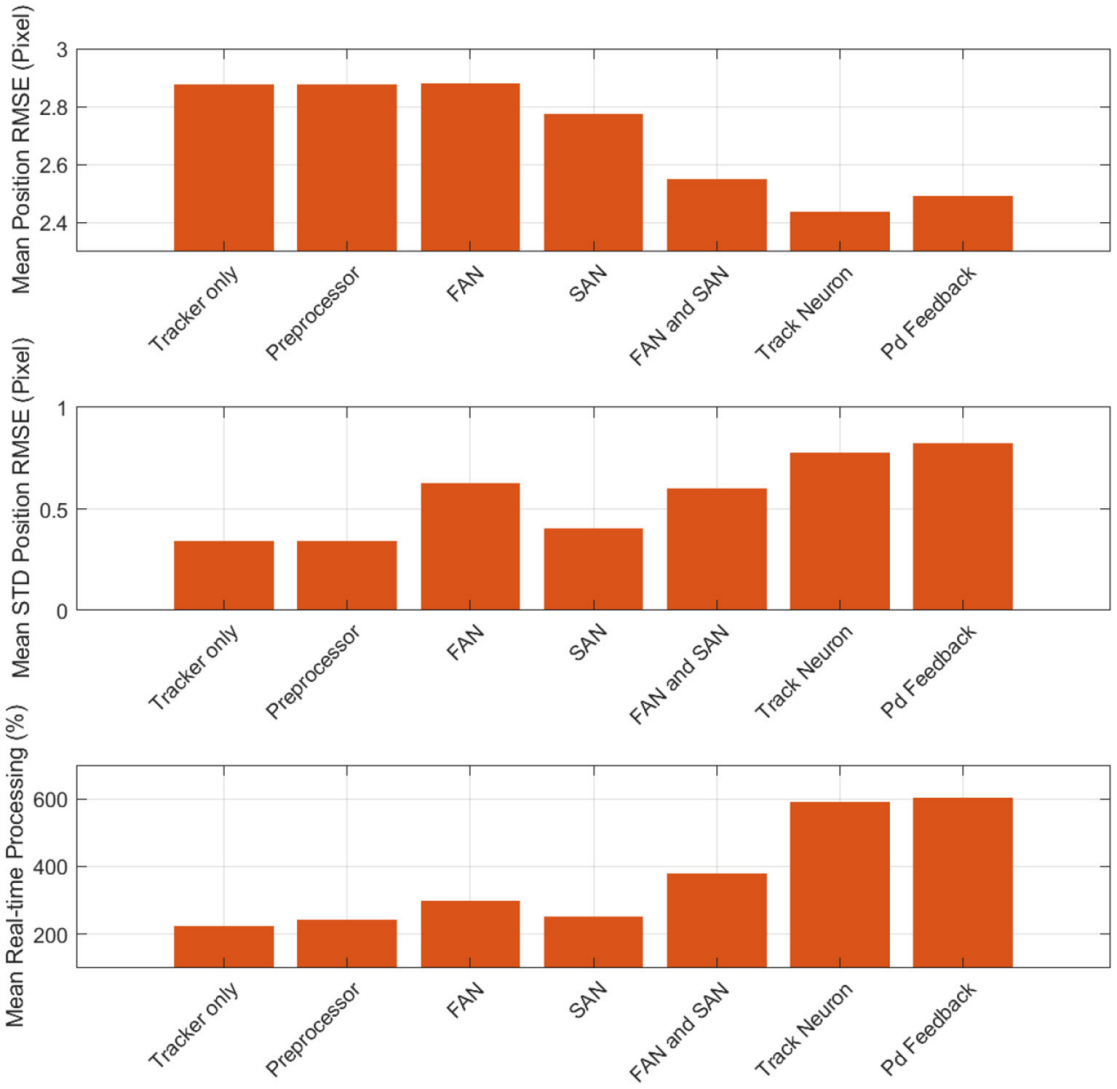
Comparison of the mean RMSE localization error (pixel), mean standard deviation of the localization error (pixel) and mean real-time processing performance across all simulated scenarios at each stage of FIESTA.

In Tables 6, 7 and Figure 14, we examine FIESTAs components and show that each stage contributes to the overall performance. By evaluating the FAN and SAN separately then together as a feature consolidator, we show quantitatively that the consolidation mechanism improves the final performance greater than the combined performance of the individual networks. In Table 6, the localization error using an isolated SAN is lower than the feature consolidator for the lowest contrast simulation in the fast and faint scenario (200 Km altitude magnitude 12). This behavior suggests that while the feature consolidator broadly improves accuracy and processing time across the 8 other scenarios, it may be filtering out too many events in the lowest contrast scenario which would otherwise be detected using longer time-scale learning in an isolated SAN. The feature consolidator and track neuron stages are shown to significantly improve the real-time processing performance despite the additional processing overhead introduced by each component. The $p_{D}^{k}$ feedback mechanism is shown to marginally reduce the performance of FIESTA while only marginally improving the real-time computational performance.

**Table 6:** Performance evaluation at each stage of FIESTA without post-processing.

**FIESTA component** **(cumulative)**	**Mean position** **RMSE (pixel)**	**Mean real-time** **processing (%)**	**Mean velocity error** **(pixel)**	**Mean GOSPA**
Tracker	2.8759	221.7626	12.6018E-06	0.8156
Preprocessor	2.8739	240.9913	12.6589E-06	0.8156
FAN	2.8779	297.2654	74.9138E-06	0.8176
SAN (no FAN)	2.7735	250.9218	22.6100E-06	0.8131
FAN and SAN	2.5491	379.7335	61.8122E-06	0.8176
Track Neuron	2.4366	588.4136	38.4575E-06	0.8180
Pd Feedback	2.4899	602.7380	40.5514E-06	0.8184

**Table 7:** Mean RMSE localization error in pixels at each stage of FIESTA across all simulated testing scenarios.

**Component Position** **RMSE (Pixel)**		**Tracker**	**Pre-processor**	**FAN**	**SAN** **(no FAN)**	**SAN and** **FAN**	**Track neuron**	**Pd** **Feedback**
**Case**								
200 Km	Mag 12	3.321	3.322	3.572	3.218	3.328	3.252	3.317
	Mag 9	2.850	2.847	2.837	2.763	2.474	2.173	2.227
	Mag 6	2.493	2.491	2.502	2.470	2.314	2.256	2.332
750 Km	Mag 12	3.401	3.402	3.509	3.257	3.156	3.140	3.213
	Mag 9	2.804	2.804	2.729	2.667	2.384	2.150	2.180
	Mag 6	2.536	2.536	2.741	2.446	2.315	2.165	2.207
2,000 Km	Mag 12	3.163	3.163	2.841	3.065	2.524	2.438	2.431
	Mag 9	2.823	2.806	2.704	2.708	2.299	2.180	2.297
	Mag 6	2.493	2.493	2.466	2.368	2.148	2.175	2.204

The performance contribution of the offline post-processor is significant as expected since targets in this linear and constant velocity leap-frog tracking task can be easily fit after tracking. In Table 8, the localization and velocity estimation error for the full FIESTA output with and without post-processing is compared to post-processed raw data and a post-processed random sample of the raw data. The number of state estimates from the full FIESTA output in each simulated observation was used to determine the sample count in the randomly sampled raw data tests. These results show as expected that the post-processed output of FIESTA has improved performance. Additionally, the post-processor alone is capable of producing reasonable accuracy in this simple STT scenario which is comparable to the raw output of FIESTA. However, the raw FIESTA output produces a lower velocity estimation error than the post-processed raw data and randomly sampled raw data. We clearly show the performance of the post-processed randomly sampled raw data is substantially poorer than the post-processed FIESTA output, which demonstrates FIESTA is correctly filtering events to produce accurate target state estimations.

**Table 8:** Comparison of the localization and velocity estimation error in the raw data and the FIESTA output when post-processed, with the lowest error (most ideal) shown in bold.

**Data source**	**Average position RMSE (pixels)**	**Average velocity RMSE (pixels per Second)**
Post-processed raw data	2.176	4.667E-04
Post-processed randomly sampled raw data	2.383	3.143E-04
Final FIESTA output	2.437	4.055E-05
Post-processed final FIESTA output	**0.211**	**3.918E-06**

The GOSPA metric recorded for each component do not vary significantly, indicating that the lowered localization error observed at each stage is not achieved at the cost of poorer overall track quality. Velocity estimation error gradually increases with all FIESTA components active, however the recorded error remains low and in the range of 1 × 10^−5^ pixels per Second (without post-processing). These error changes are due to FIESTA progressively filtering more events from the target with each new component of FIESTA, which reduces the number of data points over which the velocity can be estimated. In Figure 14, similar gradual increases in the mean standard deviation of the localization RMSE can be observed with the introduction of each component. The cause of this change is FIESTA gradually improving the localization error for a majority of simulated observations, while a small number of outlier observations (shown as outliers in Figure 13) are not similarly improving.

## 6. Discussion and Future Work

In this article, we demonstrate FIESTA as an accurate STT algorithm capable of sub-pixel position and velocity error with low variance. Evaluated using simulated event-based SSA data, these results indicate that FIESTA can successfully perform STT SSA tasks, and is a fundamental step toward MTT for SSA. With leap-frog observing, FIESTA can be used to track known or un-cued targets, and accurately estimate the target velocity using only a single transit of the RSO through the FOV.

By evaluating FIESTA on simulated data, we separate the combined performance of the proposed telescopic observing system and the EBC from the performance of FIESTA. In an optical setup with a smaller FOV, or with a higher resolution EBC, the angular sky localization error could be reduced. For example, with the latest high resolution 1280 × 720 Prophesee camera (Finateu et al., 2020), the individual pixel size is significantly smaller, which is expected to result in correspondingly smaller sky-position localization error.

We demonstrate the localization accuracy and processing time improvements provided by each component of FIESTA and show the successful combination of the FAN and SAN as a feature consolidator. However, the $p_{D}^{k}$ feedback mechanism was observed to be sub-optimal and will need improvement in future work. An individual SAN was shown to have a lower localization error than the feature consolidator on the lowest contrast scenario (200 Km at magnitude 12) but with significantly reduced real-time processing performance. Future iterations of FIESTA with a greater focus on tracking exceedingly faint targets at the cost of processing time will likely require a feature consolidator with lower learning rates.

The high-speed performance and low latency of FIESTA are highlighted by the low Target Time to Acquire (TTA) observed consistently across each simulated scenario, demonstrating FIESTA can react quickly to new targets entering the FOV. The low recorded TTA also suggests that a FIESTA-enabled SSA system could be used for closed-loop target tracking with the inclusion of an MTT algorithm to handle background astrophysical targets. Given FIESTA operates accurately and in real-time, we are confident that it can handle similar data rates from the most recent high resolution and high bandwidth HD Prophesee-Sony Sensor EBCs.

We assume a point object model for tracked targets to simplify computation. Successive iterations of FIESTA will be used investigate an extended object model to account for the spatial characteristics of targets. Extended Target Tracking (ETT) will allow FIESTA to track seemingly more extended targets with distributed structure, such as bright stars or faint targets that produce excessive wake events. ETT is expected to also reduce localization error with better estimates of the target centroid, since single events are not guaranteed to represent the target center of mass.

We demonstrate FIESTAs robust performance in multiple simulated SSA observations with the same system parameters, despite the varying dynamics found in each simulated scenario. Shown in our Supplementary Material, FIESTA requires few parameters. The authors found that of these intuitive parameters, the clutter rate and activity thresholds are the only parameters that need to tuned for different datasets if they have significantly different scene dynamics or noise properties. Tuning FIESTA is a simple process of modifying the activity thresholds to vary the number of events received by the tracker based approximately on the amount of noise in the scene. Similarly, the clutter intensity can be tuned to provide the tracker with the approximate expected number of noise measurements per unit volume.

Low state estimation error observed when evaluating the proposed asynchronous PDA in FIESTA demonstrates our successful exploration of the balance between statistically optimal and mathematically robust conventional tracking algorithms that often come with high computational cost. With accurate performance and real-time processing, FIESTA is shown to successfully balance computational speed with a robust closed-form tracking solution. The rigor and reliability of the asynchronous PDA is shown by our low localization error and correspondingly low variance. Although we ease the mathematical rigor of PDA for faster computation at the cost of optimality and information loss, we still observe low error. The proposed algorithm also strikes a balance between accuracy and latency with event-basis processing, while avoiding measurement techniques such as generating event frames with predetermined integration times. Additionally, we use our novel feature consolidation network as a solution to the low information content of an individual event by dynamically accumulating sufficient events for tracking on the event-basis. We show with confidence that we have produced an event-based processing system that makes full use of the performance and characteristics of the EBCs temporal resolution, speed, asynchronous processing and dynamic range.

While this article explores STT, we plan to explore an active RSO tracking task, where a MTT algorithm such as JPDA or GM-PHD is used to track an RSO and the apparent motion of background stars during closed-loop telescope slewing. In a MTT scenario, the event-based assumptions and constraints proposed in this article would lead to a similarly simplified one-to-many measurement-track assignment problem as opposed to many-to-many problem. In this scenario, only one measurement per scan can be associated with one of many tracks, which greatly reduces the computational cost of the assignment operation. The draw-back with this approach is that tracks are coasted more often. Coasting would occur each time a track is not associated with measurement, meaning some tracks may be coasted for *N* − 1 scans (or more in extreme cases where a target produces very few events) and be penalized more often by $\left(1-{p^{k}}_{D}\right)$, before being associated to a measurement. For the STT case, this problem is not as pronounced since it is desirable to track only the most salient target, which would ideally produce an event every tracker update interval. Maintaining this mathematical rigor and optimality becomes significantly more important when faced with MTT event-based SSA, and with higher data rates and increased clutter.

For generalized scenarios, we expect that the current STT version of FIESTA with a point target model would perform well for similar tasks with small fast moving single targets. With this point target model, performance will diminish with large extent targets. Additionally, the feature consolidator is configured with relatively small 11 × 11 neurons/contexts which limits the size of the learned features. These dimensions can be increased for larger targets at the cost of increased processing time.

MTT capabilities would be required to track targets in the presence of increased clutter targets and complex background conditions. FIESTAs rapid learning capabilities, millisecond latency and implementation of the well-established PDA tracker suggests that FIESTA is capable of tracking targets with varying motion dynamics and appearance. However, the long-term behavior of FIESTA has not yet been studied.

Target speeds consistent with RSOs in GEO are not used to evaluate FIESTA in the leap-frog scenario since the combination of the leap-frog observing method and the low apparent speed of the RSO would produce very little detectable contrast for the EBC. The limiting magnitude of the current generation three DVS EBC has been observed to be approximately 13.5.

As discussed in Section 5, the low mutual motion between the observer and a GEO target will cause a leap-frog manoeuvre to produce very little contrast in the sensor. A traditional tracking slew will produce higher contrast on a smaller region of the image plane, but it would also induce motion in the background star field, which requires an MTT algorithm. This inherently limits the current STT version of FIESTA to single target leap-frog scenarios and potentially only bright GEO. We expect the main tracking limitations are the capabilities of the EBC (limited to targets brighter than magnitude 13.5) and the brightness of the target itself, which is a function of size, geometry, material composition and sun illumination.

## 7. Conclusion

In this article, we propose and evaluate FIESTA as an accurate, and real-time tracking by detection algorithm for the novel event-based SSA paradigm. FIESTA makes full use of the EBCs capabilities by operating online and unsupervised to achieve statistically robust and closed-form tracking. The evaluated accuracy and high-speed performance indicate that FIESTA is well-suited to SSA tasks such as RSO detection, tracking, localization and orbit determination. FIESTA is demonstrated to be robust and operate with few tunable parameters. Our novel unsupervised feature consolidation networks and track neurons are highly plastic, and quickly adapt to new targets within the scene with few priors on target appearance and state dynamics. We successfully analyse the event-based paradigm shift to develop theory for the appropriate processing of event-based SSA tracking and in-frame velocity estimation. Our results show we successfully explore the trade-off between the higher spatial resolution and light intensity collection at the pixel that conventional vision sensors possess and the high temporal resolution of the EBC. This work is a fundamental step toward a MTT solution to the challenges of event-based SSA. The theory developed here precisely lays the foundation for robust closed-loop event-based tracking using rigorous closed-form solutions that are evaluated using standardized tracking metrics.

## Data Availability Statement

The datasets presented in this study can be found in online repositories. The names of the repository/repositories and accession number(s) can be found below: https://github.com/neuromorphicsystems/IEBCS.

## Author Contributions

NR conducted the investigation, developed the methodology and software, performed the validation, and wrote the original draft. DJ reviewed and edited the original draft and produced the simulated data used in this study. AJ reviewed and edited the original draft. SA, NT, GC, and AS reviewed and edited the original draft, in addition to supervising the project and contributing to the methodology. All authors contributed to the article and approved the submitted version.

## Funding

This project was funded by Western Sydney University's Strategic Research Initiative. Some of the authors were supported by AFOSR grant no. FA9550-18-1-0471.

## Conflict of Interest

The authors declare that the research was conducted in the absence of any commercial or financial relationships that could be construed as a potential conflict of interest.

## Publisher's Note

All claims expressed in this article are solely those of the authors and do not necessarily represent those of their affiliated organizations, or those of the publisher, the editors and the reviewers. Any product that may be evaluated in this article, or claim that may be made by its manufacturer, is not guaranteed or endorsed by the publisher.

## Footnotes

^1^Data available from https://github.com/neuromorphicsystems/IEBCS.
