# Supplementary material for: Real-Time Event-Based Unsupervised Feature Consolidation and Tracking for Space Situational Awareness
Source: Front Neurosci. 2022 May 6;16:821157. doi: 10.3389/fnins.2022.821157 (PMC9120364; doi:10.3389/fnins.2022.821157)
Supplement: Supplementary file 1 [file Data_Sheet_1.PDF]

Supplementary Material

1 SUPPLEMENTARY ALGORITHMS AND PSEUDO-CODE

We present the Pseudo-code for the Feature Extraction using Adaptive Selection Thresholds (FEAST) algorithm in Algorithm 1, the asynchronous Probabilistic Data Association (PDA) tracker in Algorithm 2 and Fast Iterative Extraction of Salient targets for Tracking Asynchronously (FIESTA) in Algorithm 3. The parameters listed in these Algorithms are detailed in Tables S1 and S2.

Algorithm 1: The FEAST algorithm

```
Result: Neuron spiking flag, winning neuron n_win and weight W_win, and spiking event u_i
Initialise all neurons n with random weights;
if Event-context π_e^r received then
    Lower neuron spiking flag;
    for i = 0; Neuron n_i ; i++ do
        Calculate cosine similarity D_N for π_e^r and W_i;
    end
    Winning neuron index n_win = argmax(D_N);
    for i = 0; Neuron n_i ; i++ do
        if Distance within neuron threshold D_i ≥θ_i then
            if Neuron n_i is winning neuron, n_i == n_win then
                Raise neuron spiking flag;
                Update neuron: W_i = (1 − η)W_n + ηπ_e^r(u_i, t);
                Raise selectivity, lower θ_i by Δi;
            end
        else
            Lower selectivity, raise θ_i by Δe;
        end
    end
end
```

Algorithm 2: FIESTA tracking algorithm component

Result: Track State Estimation $\rho(x_k|Z_{1:k-1})$

if *Event* $\mathbf{u}_i$ *Received* **then**
- ++k;
- **if** *!Tracks.empty()* **then**
  - ++Track Age;
  - **if** $\epsilon_k^{\theta} S_k^{-1} \epsilon_k^{\theta} \leq \gamma$ *and* ${W_{win}}^{FAN}$ *Spikes Track Neuron* **then**
    - Perform Async PDA Update;
    - ++n Associations;
    - Last Measurement = 0;
    - **if** *n Associations* $\geq A_{confirm}$ **then**
      - Track == Confirmed;
      - Raise p_D^k of N_{SAN} by $p_D +$;
    - **else**
      - Track == Tentative
    - **end**
  - **else**
    - Track == Coasted;
    - Perform Coasted Async PDA Update;
    - ++Last Measurement;
    - **if** *Last Measurement* $\geq A_{miss}$ *and Track Likelihood* $\leq P_{del}$ **then**
      - Track == Deleted;
      - Lower p_D^k of N_{SAN} by $p_D -$;
    - **end**
  - **end**
- **else**
  - **if** *Mutual Event-Tracks Dist* $D_{euc} \leq \alpha$ **then**
    - *Initialise Tentative Track at* $\mathbf{u}_i$*;*
    - *++Track Age;*
    - *Last Measurement = 0;*
  - **end**
- **end**

end

Algorithm 3: FIESTA algorithm

```
Result: Salient target state estimation ρ(x_k|Z_{1:k−1})
if Event u_i Received then
    Calculate time surface Π_e(u_i, t);
    Extract π_e(u_i, t)
    if −δ_π ≤ Σ_{i,j} π_e(u_i, t) ≥ δ_π then
        Input π_e(u_i, t) to Fast Adapting Network (FAN) network;
        if FAN network spikes; then
            if −δ_FAN ≥ Σ_{i,j} W_win^FAN ≤ δ_FAN then
                Input W_win^FAN to Slow Adapting Network (SAN) network;
                if SAN network spikes; then
                    Calculate spiking W_win^SAN activity;
                    if −δ_SAN ≤ Σ_{i,j} W_win^SAN ≥ δ_SAN then
                        if −δ_{Track−neuron} ≥ Σ_{i,j} W_{Track−neuron} ≤ δ_{Track−neuron} then
                            Input u_i, W_win^FAN to PDA tracker;
                        end
                    end
                end
            end
            Output all track states x_k and time-stamp t;
        end
    end
end
```

Component	Parameter	Value
Feature Consolidator and Learning	Time Surface ($\Pi_e(u_i, t)$) decay constant (τ)	$5 \times 10^4\ \mu s$
	Event-Context ($\pi_e(u_i, t)$) Dimensions	11×11
	Event-Context Activity Threshold (δ_π)	≥0.5
	FAN Neuron Activity Threshold (δ_{FAN})	≥4
	SAN Neuron Activity Threshold (δ_{SAN})	≤10.65
	Track Neuron Activity Threshold (δ_{TN})	≤12.5
Tracker	Elliptical Gating Threshold (γ)	-9.5
	Mutual Track-Measurement Euclidean Distance Threshold (α)	20
	Field of View (Pixels)	346×240
	Track Likelihood Deletion Threshold (P_{del})	-12
	Number of Associations to Confirm Track ($A_{confirm}$)	5
	Initial Detection Probability (P_{D0})	0.75
	Detection Probability Raising Factor (P_{D+})	0.24
	Detection Probability Reduction Factor (P_{D-})	0.25
	Detection Probability (P_{Dk}) Limits	0.5 - 0.99
	Clutter Rate ($\bar{\lambda}_k$)	3×10^1
	Number of Consecutive Missed Associations (A_{miss})	10

Table S1. Parameters used by FIESTA during computation. All values are constant across processing and all evaluation scenarios. The clutter rate and activity thresholds are the only parameters here that are tuned for different scene dynamics, EBCs and noise properties.

2 SUPPLEMENTARY TABLES AND FIGURES

FIESTA operates robustly across multiple simulated Space Situational Awareness (SSA) observations with the same system parameters, despite the varying dynamics found in each simulated scenario. Here we show these few parameters in Tables S1 and S2. The authors found that of these intuitive parameters, the clutter rate and activity thresholds are the only parameters that need to tuned for different datasets if they have significantly different scene dynamics or noise properties.

Component	Parameter	Value
Fast Adapting Network (3×3) (FAN)	Learning Rate (η)	1×10^{-2}
	Initial Selection Threshold	1×10^{-6}
	Selection Threshold Rise Rate	1×10^{-3}
	Selection Threshold Fall Rate	1×10^{-1}
Slow Adapting Network (3×3) (SAN)	Learning Rate (η)	5×10^{-4}
	Initial Selection Threshold	1×10^{-11}
	Selection Threshold Rise Rate	1×10^{-3}
	Selection Threshold Fall Rate	1×10^{-1}

Table S2. Network configuration and parameters of the FAN and SAN networks in FIESTA. All parameters are constant across processing and all evaluation scenarios.
